# Tumor Lipids of Pediatric Papillary Renal Cell Carcinoma Stimulate Unconventional T Cells

**DOI:** 10.3389/fimmu.2020.01819

**Published:** 2020-08-20

**Authors:** Nadine Lehmann, Claudia Paret, Khalifa El Malki, Alexandra Russo, Marie Astrid Neu, Arthur Wingerter, Larissa Seidmann, Sebastian Foersch, Nicole Ziegler, Lea Roth, Nora Backes, Roger Sandhoff, Joerg Faber

**Affiliations:** ^1^Department of Pediatric Hematology/Oncology, Center for Pediatric and Adolescent Medicine, University Medical Center of the Johannes Gutenberg-University Mainz, Mainz, Germany; ^2^University Cancer Center (UCT), University Medical Center of the Johannes Gutenberg-University Mainz, Mainz, Germany; ^3^Institute of Pathology, University Medical Center of the Johannes Gutenberg-University Mainz, Mainz, Germany; ^4^Lipid Pathobiochemistry, German Cancer Research Center, Heidelberg, Germany

**Keywords:** pediatric papillary renal cell carcinoma, TILs, unconventional T cells, CD1d, lipid antigens

## Abstract

Papillary renal cell carcinoma (PRCC) is a rare entity in children with no established therapy protocols for advanced diseases. Immunotherapy is emerging as an important therapeutic tool for childhood cancer. Tumor cells can be recognized and killed by conventional and unconventional T cells. Unconventional T cells are able to recognize lipid antigens presented via CD1 molecules independently from major histocompatibility complex, which offers new alternatives for cancer immunotherapies. The nature of those lipids is largely unknown and α-galactosylceramide is currently used as a synthetic model antigen. In this work, we analyzed infiltrating lymphocytes of two pediatric PRCCs using flow cytometry, immunohistochemistry and qRT-PCR. Moreover, we analyzed the CD1d expression within both tumors. Tumor lipids of PRCC samples and three normal kidney samples were fractionated and the recognition of tumor own lipid fractions by unconventional T cells was analyzed in an *in vitro* assay. We identified infiltrating lymphocytes including γδ T cells and iNKT cells, as well as CD1d expression in both samples. One lipid fraction, containing ceramides and monoacylglycerides amongst others, was able to induce the proliferation of iNKT cells isolated from peripheral blood mononuclear cells (PBMCs) of healthy donors and of one matched PRCC patient. Furthermore, CD1d tetramer stainings revealed that a subset of iNKT cells is able to bind lipids being present in fraction 2 via CD1d. We conclude that PRCCs are infiltrated by conventional and unconventional T cells and express CD1d. Moreover, certain lipids, present in pediatric PRCC, are able to stimulate unconventional T cells. Manipulating these lipids and T cells may open new strategies for therapy of pediatric PRCCs.

## Introduction

In contrast to adults, where renal cell carcinoma accounts for two to three percent of all malignancies (1), pediatric renal cell carcinoma is an extremely rare entity. Only 1.8–6.3% of all malignant kidney tumors in children are renal cell carcinomas (2, 3) with papillary renal cell carcinoma (PRCC) being the second most common type (4, 5). Selle et al. and Rao et al. studied all kidney cell carcinomas in childhood and adolescence from 1980 to 2005 in Germany and from 1989 to 2008 in a hospital in China. Among them, 34 PRCCs were listed. Despite the good prognosis of this disease, a total of 5 patients have died of their disease (6, 7). Currently, the treatment of choice is a complete surgical resection with no established therapeutic protocol for progressive disease. Due to the mortality of patients with advanced disease, it is of high interest to characterize the biology of pediatric PRCCs and subsequently provide new options for therapy.

The immune system is crucial for the suppression of cancer development and the composition of tumor-infiltrating lymphocytes (TILs) is being widely investigated as prognostic and predictive biomarker (8, 9). In adult PRCCs, the highest amount of TILs are T cells (10). However, their prognostic role is controversial because, in contrast to other entities, high density of infiltrating CD8^+^ T cells is associated with poor prognosis (8, 11–13). Considering pediatric tumor entities, little is known about the frequency and immunophenotype of TILs and no data is available for pediatric PRCCs. Infiltration of T cells is already described in medulloblastoma, neuroblastoma, Wilms tumors, osteosarcoma and Ewing sarcoma (14–19). Here, the characterized cells were mainly conventional T cells. An infiltration of γδ T cells and of invariant NKT (iNKT) cells was detected in medulloblastoma (14) and in neuroblastoma (16), respectively. γδ T cells and iNKT cells are unconventional T cells which are not restricted to the major histocompatibility complex (MHC) and recognize lipids, small metabolic molecules and specially modified peptides as antigens (20). Both unconventional T cell subsets are able to interact with cells from the innate and adaptive immune system, which subsequently can attack tumor cells (21, 22). For example, iNKT cells as well as γδ T cells can kill tumor cells via the perforin-granzyme-pathway (22, 23). A subgroup of γδ T cells, Vδ1^+^ γδ T cells, and iNKT cells are able to recognize lipid antigens presented via CD1d which belongs to the family of MHC class I-related CD1 molecules (24). Various lipid antigens can be presented via CD1d, including sphingolipids and glycerolipids which are generally endogenous lipids. In addition, exogenous lipids, microbial antigens, hydrophobic peptides and small non-lipid molecules can be presented by CD1d (24). Nevertheless, the nature of those lipids is largely unknown. The synthetic sphingolipid α-galactosylceramide (α-GalCer), isolated from a marine sponge, as well as endogenous α-GalCer and α-GalCer derived from bacteria like *B. fragilis* showed the ability to stimulate iNKT cells (25–28). Moreover, Uldrich et al. investigated that also Vδ1^+^ γδ T cells can be activated by α-GalCer (29).

Here, we aimed to characterize the landscape of infiltrating T cells and the CD1 expression of PRCCs. Furthermore, we analyzed the potential of tumor own lipid fractions to stimulate unconventional T cells.

## Materials and Methods

### Patients and Material

Blood and tumor samples were obtained from two male patients (sample 277 and 288) with papillary renal cell carcinoma diagnosis. At the time of diagnosis, the patients were 8 and 10 years of age, respectively. As control, normal kidney tissue from patients (sample 98, 181, and 206) aged respectively two, 15 and 1 year(s) of age with Wilms Tumor diagnosis were used as control samples. This study was performed in agreement with the declaration of Helsinki. Patients' legal guardians gave their informed consent for the scientific use of surplus material. Buffy Coats from healthy donors were obtained from the Transfusion Center of the University Medical Center of the Johannes Gutenberg-University in Mainz. Formalin-fixed, paraffin-embedded (FFPE) tissue samples were kindly provided by the tissue bank of the University Medical Center Mainz in accordance with the regulations of the tissue biobank and the approval of the local ethics committee.

### Isolation of Tumor-Infiltrating Lymphocytes

Fresh and native tumor material was minced into small pieces with a scalpel, continued by mechanical dissociation with GentleMACS Dissociator (Miltenyi Biotec). Mechanical dissociation was followed by enzymatical dissociation with 400 μl Liberase™ Research Grade (Cat. No. 5401127001, Roche), 30 U/ml DNase I (Cat. No. D5025, Sigma-Aldrich) and 3.6 ml HBSS Medium (Cat. No. 14170-088, Life Technologies). Tumor-infiltrating lymphocytes were isolated using Percoll density centrifugation. For that, 4 ml of 37% Percoll were layered under 4 ml of 30% Percoll dilution. Cells were resuspended in 70% Percoll and placed underneath the 37% Percoll layer. After centrifugation, the immune cells were located in the interphase between the 30 and 37% layer. Cells were transferred into a new tube, washed twice with PBS and stored at −80°C until further analysis.

### Flow Cytometry

Immunostaining of isolated tumor-infiltrating lymphocytes was performed with following antibodies: CD3-ECD (Cat. No. A07748), CD4-PC5.5 (Cat. No. B16491), CD8-Pacific Blue (Cat. No. B49182), CD45-APC (Cat. No. IM2473), Anti-TCR Pan γ/δ-FITC (Cat. No. B49175) (all Beckman Coulter), Anti-iNKT-PE-Vio770™ (Cat. No. 130-104-110), Anti-TCR Vd1-APC-Vio770™ (Cat. No. 130-100-521) and Anti-TCR Vd2-VioGreen™ (Cat. No. 130-106-653) (all Miltenyi Biotec). Cells were resuspended in 100 μl PBS and incubated with 10 μl of each antibody in the dark for 15 min. Subsequently, cells were washed twice with PBS and resuspended in PBS-BSA for flow cytometric analysis on Navios Flow Cytometer (Beckman Coulter Life Sciences). Further analyses were performed with FlowJo V10 (Tree Star Inc.).

### RNA Extraction and cDNA Synthesis

60–70 mg cryopreserved tumor tissue was co-administered with a stainless steel bead and 1 ml QIAzol (Cat. No. 79306, Qiagen) in a 2 ml microcentrifuge tube and disrupted using Qiagen TissueLyser II. RNA extraction was performed using RNeasy Lipid Tissue Kit (Cat. No. 74804, Qiagen) according to manufacturer's instructions. cDNA was synthesized from RNA using PrimeScript RT Reagent Kit with gDNA Eraser (Cat. No. RR037B, Takara Bio). Quantification was performed with Qubit™ 3.0 Fluorometer (Thermo Fisher Scientific).

### qRT-PCR

Quantitative real time PCR was performed with the Light Cycler® 480 Detection System and Software (Roche) using PerfeCTa® SYBR® Green FastMix® (Cat. No. 95072, Quanta BioSciences). As an endogenous reference, the results were normalized to *HPRT*. The following primers were used: *CD45*: Forward 5′-TGATAAGACAACAGTGGAGAAAGG-3′, Reverse 5′-CTGTCACAAATACTTCTGTGTCCA-3′; *HPRT*: Forward 5′-TGACACTGGCAAAACAATGCA-3′, Reverse 5′-GGTCCTTTTCACCAGCAAGCT-3′; *TCR* γ: Forward 5′-AACGGTGCCAGAAAAGTCAC-3′, Reverse 5′-TGTCTTTGGGATCCATTGTG-3′ (30); *TCR* δ: Forward 5′-CTGTGCACTCCACTGACTTTG-3′, Reverse 5′-GGGTTTATGGCAGCTCTTTG-3′ (30); *TCR V*α*24J*α*18*: Forward 5′-CCTCCCAGCTCAGCGATTC-3′, Reverse 5′-TATAGCCTCCCCAGGGTTGA-3′ (31); *TCR V*δ*1*: Forward 5′-TCGCCTTAACCATTTGAGCC-3′, Reverse 5′-AACGGATGGTTTGGTATGAGGT-3′ (32); *TCR V*δ*2*: Forward 5′-GAGAACCAGGCTGTACTTAAGATCCTT-3′, Reverse 5′-TGACGAAAACGGATGGTTTG-3′ (32).

### Immunohistochemistry

Sections of FFPE tissue samples were deparaffinized in xylene, hydrated in a descending ethanol series and washed in Aqua dest. Demasking of the sections was performed with (1×) Target Retrieval Solution (Cat. No. S170084-2, Dako) for TCR δ and CD45 stainings or Tris EDTA pH 9.0 demasking buffer (10 mM Tris, 1 mM EDTA, 0.05% Tween®20 in Aqua dest., pH 9.0) for CD1d staining at 95°C for 40 min in a decloaking chamber (NxGen DC Modul, Biocare Medical). After three washing steps with PBS-T, the sections were treated with Peroxidase Blocking Solution (Cat. No. S202386-2, Dako). Subsequently, the samples were washed with Aqua dest. and PBS-T and blocked with Vector Horse Serum 2.5% (Cat. No. S-2012, Vector Laboratories) for 30 min. Sections were incubated with monoclonal mouse anti-CD45 (Leucocyte Common Antigen, Clones 2B11 + PD7/26, Cat. No. M0701, Dako), anti-TCR δ (H-41, Cat. No. sc-100289, Santa Cruz) and anti-CD1d ([NOR3.2 (NOR3.2/13.17)], Cat. No. ab11076, Abcam) in a humidification chamber for 1 h at room temperature. The chosen dilutions were: 1:100 for anti-CD45, 1:50 for anti-TCR δ and 1:150 for anti-CD1d. After incubation with secondary anti-mouse IgG antibody (ImmPRESS™, Cat. No. MP-7402, Vector Laboratories) for 30 min, several washing steps were performed and the sections were stained with 3,3′-Diaminobenzidine (Cat. No. SK-4100, Vector Laboratories) to label positive structures. Accordingly, the samples were washed, counterstained with Hematoxylin (Cat. No. S3309, Dako) and dehydrated with ascending ethanol series.

### Quantitative Analysis of IHC Stainings

Stained slides were digitalized using NanoZoomer-Series Digital slide scanner (Hamamatsu Photonics). DAB-positive areas were quantified using HALO® platform from Indica Labs. Hematoxylin was used as nuclear counter stain. Gross tumor areas were annotated by a pathology expert and image analysis was performed with the same predefined settings for all slides. The percentage of positive or negative cells of all cells within the region was used for downstream analysis.

### Isolation and Fractionation of Tumor Own Lipids

50–60 mg of lyophilized tumor tissue were homogenized in 80 μL Aqua dest. and 800 μL methanol (−20°C) twice for 2 min at 25 Hz in a TissueLyser II (Qiagen). The total volume of the homogenate was determined. An aliquot of 20 μl was used to determine the total protein concentration with a standard BCA assay. For lipid extraction, 827 μL of the homogenate were mixed with 727 μL chloroform and incubated 15 min at 37°C using 3 times 3 min ultrasound. Samples were centrifuged for 1 min at 13,000 rpm and supernatant was collected. The residual pellet was reextracted with 1.5 mL chloroform:methanol:water (10:10:1) as before and resulting supernatant was collected with the first supernatant. Finally, the residual pellet was re-extracted with chloroform:methanol:water (30:60:8) as before and resulting supernatant was pooled with the first two supernatants. The lipid raw extract was dried with a gentle nitrogen stream at 37°C.

Aminopropyl columns (100 mg stationary phase) were preconditioned with 2 mL hexane and the lipid fractions were dissolved in 200 μL chloroform per 60 mg tissue wet weight using ultrasound. A volume of 200 μL of dissolved lipids was loaded on a column and the run-through was collected as fraction 0. The column was then eluted with 1.4 mL ethylacetate:n-hexane (15:85) (→ fraction 1), 1.6 mL chloroform:methanol (23:1) (→ fraction 2), 1.8 mL diisopropylether:acetic acid (98:5) (→ fraction 3), 1.9 mL acetone:methanol (9:1,35) (→ fraction 4), 1.9 ml chloroform:methanol (2:1) (→ fraction 5) and 1.9 mL chloroform:methanol:3,6 M aqueous ammonium acetate (30:60:8) (→ fraction 6). Aliquots of each fraction corresponding to 8 mg tissue wet weight were dried subsequently with a gentle nitrogen-stream at 37°C.

### Analysis of Lipids by Reversed Phase Liquid Chromatography-Coupled Tandem Mass Spectrometry

Aliquots corresponding to 8 μg total protein were mixed with internal lipid standards and dissolved in 1 ml 95% methanol for analysis by LC-MS^2^ using an Acquity I-class UPLC and a Xevo TQ-S “triple-quadrupole” instrument, both from Waters. Using a CSH C18 column (2.1 mm × 100 mm; 1.7 μm, Waters), lipids were measured in reversed phase-LC (RPLC) mode with a gradient between 57% solvent A (50% methanol) and 99% solvent B (1% methanol, 99% isopropanol), both containing 0.1% formic acid and 10 mM ammonium formate as additives. From the samples, 10 μL were injected for measurement. Lipids analyzed were detected by multi-reaction monitoring (MRM) and data of each lipid class were normalized to the fraction containing the respective maximum for this class. Internal standards for 1-O-acylceramides (1-OAC), ceramides (Cer), hexosylceramides (HexCer), sphingomyelins (SM) and phosphatidylcholines (PC) had been added to the lipid extracts according to (33, 34), allowing for absolute quantification. For all other lipids, the absolute detected signal intensities in arbitrary units were used to perform relative quantification.

### *In vitro* Assay for Potential Immunogenic Lipid Fractions

PBMCs from healthy donors and from one PRCC patient (277) were isolated by Ficoll density gradient centrifugation. The separations of iNKT, γδ T cells and CD14^+^ cells were performed by immunomagnetic separation with MicroBeads (Cat. No. 130-094-842, 130-092-892, 130-050-201 Miltenyi Biotec). First, 3 × 10^4^ to 1 × 10^5^ iNKT and γδ T cells depending on cell distribution and frequency of each donor, were cultured with RPMI complete medium supplemented with 20 U/ml IL-2 (PeproTech) in 24-well plates. *In vitro* assays with patient-derived cells were performed with 1-1.5 × 10^3^ iNKT cells and 96-well plates were used. For the differentiation into immature dendritic cells (DCs), CD14^+^ cells were cultured in RPMI complete medium with 100 ng/ml IL-4 and 50 ng/ml GM-CSF (both PeproTech) for 5 days. Immature DCs were incubated with 200 ng/ml of the control antigen α-Galactosylceramide (Cat. No. KRN7000, Funakoshi) or with the respective lipid fractions corresponding to 8 mg tumor wet weight at 37°C, 5% CO_2_ for 24 h. DMSO-treated DCs served as negative control (0.2 μl DMSO/1 ml medium). Untreated iNKT and γδ T cells were cultivated with the same conditions to demonstrate whether DMSO-treated DCs have any effect on the respective T cell population. After one washing step, lipid-loaded DCs were co-cultivated with iNKT or γδ T cells (ratio 2:1) in fresh RPMI complete with 100 U ml IL-2. There were two more restimulations with new lipid-loaded DCs on day 9 and 12. On day 15, the cells were harvested for flow cytometric analysis. For that, harvested cells were resuspended in 300 μl PBS-BSA and measured with Navios Flow Cytometer. Acquisition time was 130 s. The value of DMSO-vehicle control was defined as 100%. Population size of untreated T cells and lipid-stimulated T cells were calculated relative to DMSO control. Those fractions inducing proliferation >30% were considered as immunogenic.

### CD1d Tetramer Preparation

Empty, PE-conjugated CD1d-tetramers were obtained from ProImmune (Cat. No. D000-2X). As positive control, 1.5 μl tetramers were loaded with a 100-fold molar excess of α-Galactosylceramide (KRN700). Tumor lipid fraction aliquots of 2 mg wet weight were dissolved in 0.8 μl DMSO and 999.2 μl PBS with 0.05% Tween® 20 and were incubated in supersonic water bath at 80°C for 2 min. 1.5 μl CD1d-tetramers with 0.8 μl DMSO and 999.2 μl PBS served as lipid-unloaded vehicle-control. Subsequently, 1 ml of each fraction was incubated with 1.5 μl CD1d tetramer. Lipid-loaded tetramers were incubated overnight at room temperature in the dark. iNKT cells from two healthy donors were separated by immunomagnetic anti-iNKT microbeads (one separation step). CD1d staining was performed according to manufacturer's instructions. Shortly, 3 × 10^5^ iNKT cells were incubated with lipid-loaded CD1d tetramers. Antibody staining was performed with CD3-ECD, CD19-A700 (Cat. No. B49212, Beckman Coulter). Flow cytometric analyses were performed using a Navios Flow Cytometer (Beckman Coulter Life Sciences). Further analyses were performed with FlowJo V10 (Tree Star Inc.).

### Statistical Analyses

Statistical analysis of the *in vitro* assays was performed using the non-parametric Kruskal-Wallis test. Statistical significance of differences between lipid concentrations of tumor and normal samples was calculated using student's *t*-test.

## Results

### Pediatric PRCCs Are Infiltrated by Conventional and Unconventional T Cells

**Figure 1:**
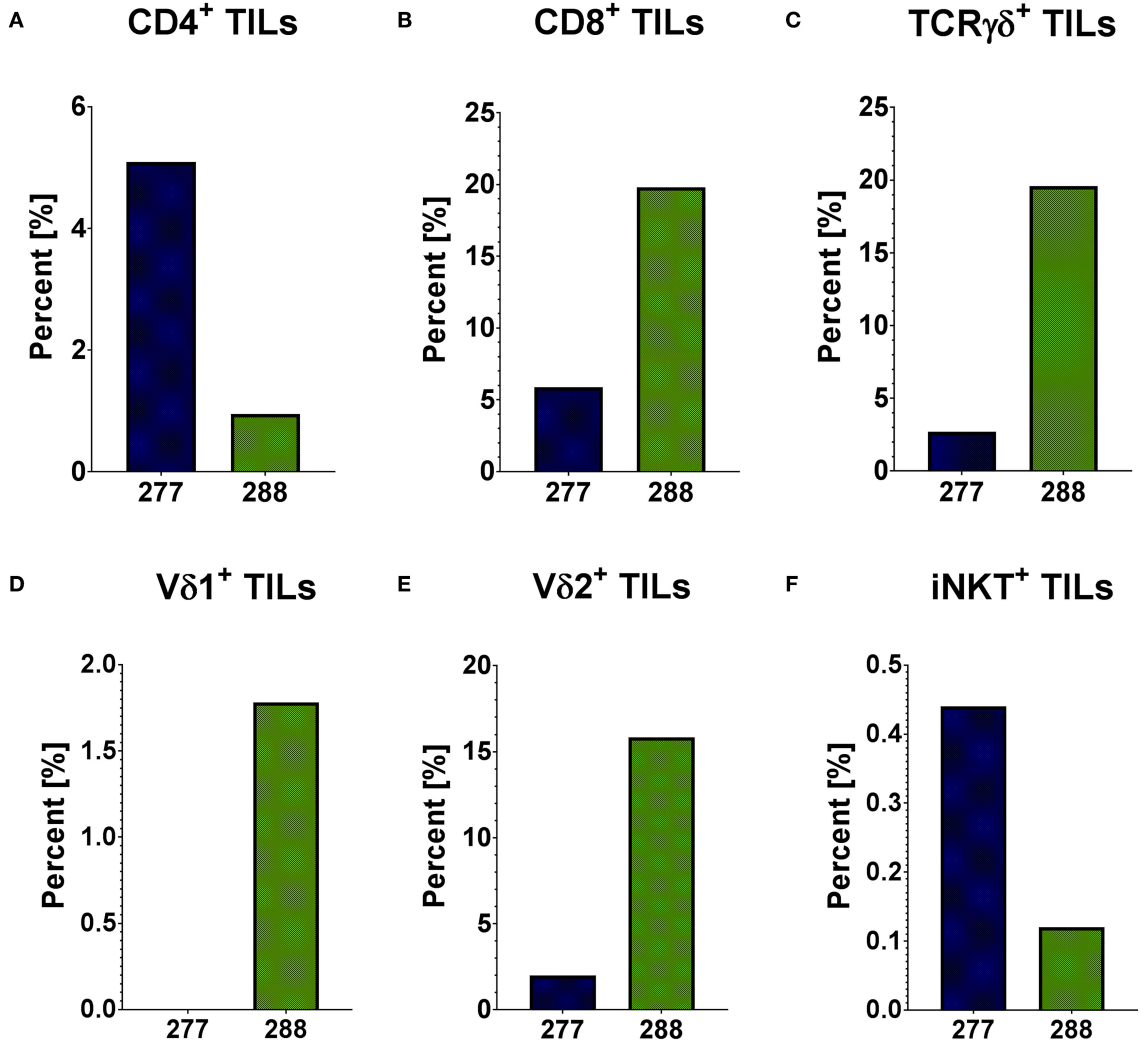
Distribution of infiltrating T cells in PRCC tumor samples. Flow cytometric analysis was performed using specific antibodies against CD4, CD8, TCRγδ, Vδ1, Vδ2 and iNKT cell receptor. The distribution of different subpopulations within the lymphocyte population is shown. Both PRCC samples are infiltrated by conventional (CD4^+^ and CD8^+^) and unconventional (γδ T-cells and iNKT-cells) T-lymphocytes. There is no infiltration of Vδ1^+^ T-cells in sample 277. **(A)** CD4^+^ T cells; **(B)** CD8^+^ T cells; **(C)** TCRγδ^+^ T cells; **(D)** Vδ1^+^ T cells; **(E)** Vδ2^+^ T cells; **(F)** iNKT cells.

In order to analyze the immunobiology of pediatric PRCCs, the different T cell subpopulations within the lymphocyte population of two patient samples were quantified by flow cytometry (gating strategy in Supplemental Figure 1). In sample 277 and 288, 1.92 and 2.00% of analyzed cells were lymphocytes, respectively. Both PRCC samples were infiltrated by conventional and unconventional T cell populations with different proportions. Sample 277 showed a higher proportion of CD4^+^ cells with 5.09% of all lymphocytes compared to sample 288 with 0.95% (Figure 1A). In contrast, sample 288 contained a higher amount of CD8^+^ cells (19.79% of all lymphocytes) than sample 277 with 5.89% (Figure 1B). Concerning the infiltration of γδ T cells, a 7-fold higher expression of the γδ T cell receptor was detected in sample 288 with 19.59% of all lymphocytes compared to 2.69% in patient sample 277 (Figure 1C). In order to distinguish the individual subpopulations of γδ T cells, stainings for the different δ chains, Vδ1 chain as well as Vδ2 chain of the γδ T cell receptor were performed. In sample 277, no Vδ1^+^ cells were detected, while in sample 288 1.78% Vδ1^+^ cells could be identified (Figure 1D). A Vδ2^+^ cell population was detected in both samples (277: 2.0%; 288: 15.84%) (Figure 1E). To further characterize the infiltration of unconventional T cells, it was investigated whether both samples were infiltrated by iNKT cells with the specific variant TCR Vα24Jα18 chain. iNKT cells were identified in both samples of pediatric PRCC. Sample 277 was infiltrated by 0.44% and sample 288 by 0.12% iNKT cells (Figure 1F). Flow cytometric analysis of the peripheral blood of the two patients confirmed that iNKT (patient 277) and γδ T cells (patient 288) are enriched in the tumor samples (Supplemental Figure 2).

**Figure 2:**
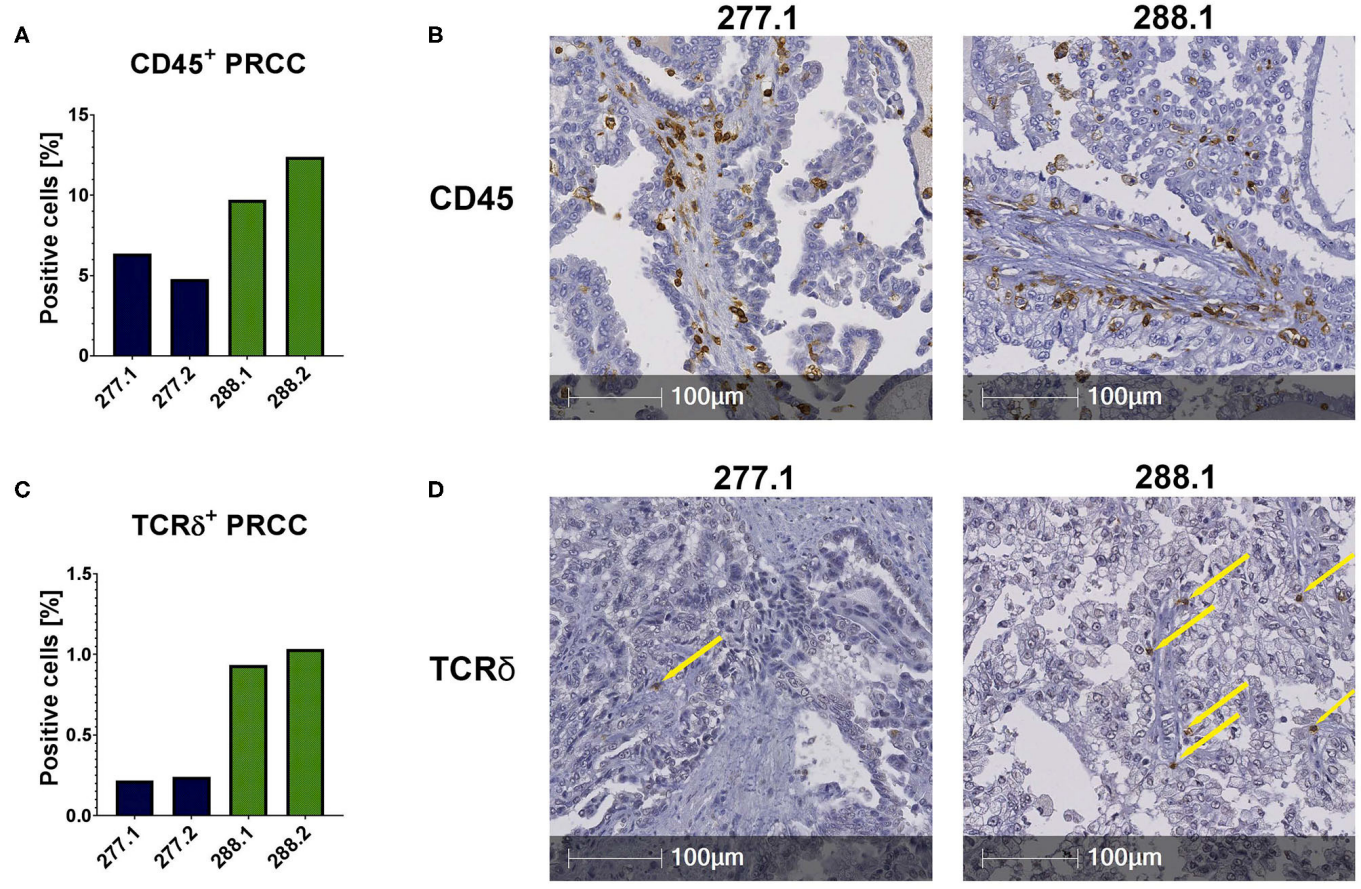
IHC stainings of PRCC tumor samples. IHC stainings were performed using specific antibodies against CD45 and TCRδ. Staining with DAB identified positive cells, nuclear counterstaining was performed using hematoxylin. Of each case two different sections were stained and analyzed (277.1, 277.2, 288.1, and 288.2). Tumor area was defined and positive cells within this area were quantified using HALO software. **(A)** Quantitative analysis of CD45^+^ labeled cells. Both samples are infiltrated by CD45^+^ cells. Sample 288 displays a higher amount of CD45^+^ cells than 277. **(B)** Anti-CD45 IHC stainings (samples 277.1 and 288.2). **(C)** Quantification of TCRδ^+^ cells. More TCRδ^+^ cells are detectable in sample 288. **(D)** Anti-TCRδ IHC stainings. Sample 288.1 shows more infiltrating TCRδ^+^ cells than sample 277.1. Yellow arrows indicate positive cells.

**Figure 3:**
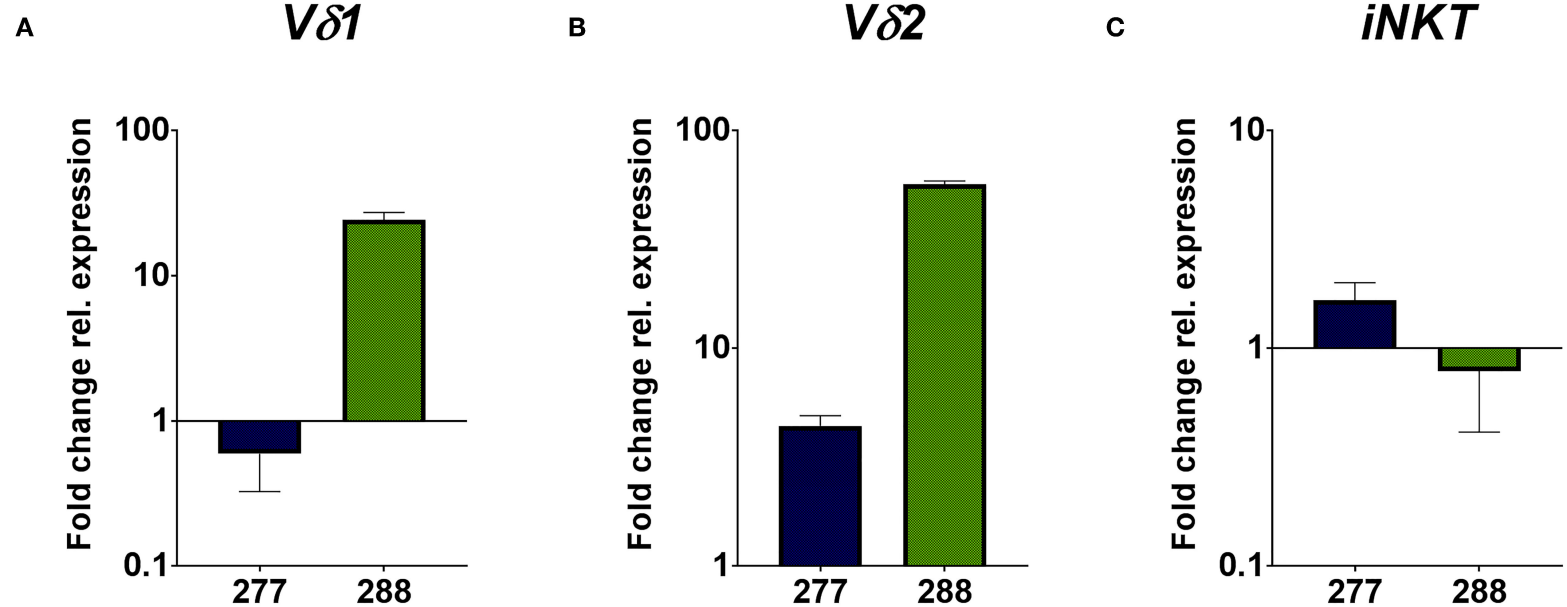
qRT-PCR analysis of unconventional T cell-related genes. Gene expression analysis was performed by qRT-PCR method. After normalization to the housekeeping gene *HPRT1*, expression quantification was calculated with the 2^−ΔΔ*ct*^ method. Fold change of relative expression was calculated relative to pediatric normal kidney tissue. Analysis was performed in triplicates. **(A)** Expression analysis of unconventional T cell-related genes show a higher expression of *V*δ*1* in sample 288, whereas no expression of *V*δ*1* is detectable in sample 277. **(B)** Expression analysis of *V*δ*2* show a higher fold change in sample 288 than in sample 277. **(C)** Expression analysis of iNKT-related *TCRV*α*24J*α*18* gene display the tendency of higher expression in sample 277 than in 288.

The flow cytometric data was corroborated by immunohistochemical stainings of two samples of each PRCC patient. For that, sections were stained for CD45 and the Vδ chain of the γδ T cell receptor. Considering the mean value of each patient, sample 277 contained 5.59% CD45-positive cells within the tumor area and sample 288 11.06% CD45-positive cells (Figures 2A,B). Moreover, we identified TCRδ^+^ cells in both samples within the tumor area. In sample 277, 0.23% were TCRδ^+^ and in sample 288 0.98% (Figures 2C,D). Because no antibody for IHC analysis is established for the detection of Vδ1, Vδ2 and iNKT cells so far, we validated the flow cytometry data by qRT-PCR. Sample 288 showed a 24-fold higher expression of *V*δ*1* than control tissue while sample 277 did not express *V*δ*1* (Figure 3A). This is consistent with our flow cytometric data that detected no *V*δ*1* T cells in sample 277 (Figure 1D). The higher *V*δ*2* expression was found in sample 288 (Figure 3B). iNKT cell marker expressions were poorly assessable due to low frequency, but with tendency to a higher expression of *V*α*24J*α*18* in sample 277 (Figure 3C).

In conclusion, our data indicate infiltration of conventional CD4^+^ and CD8^+^ T cells in sample 277 and 288. Moreover, we identified infiltration of unconventional T cells in both samples, whereas sample 277 was not infiltrated by Vδ1 T cells.

### Strong CD1d Expression Is Present in Both Pediatric PRCC Samples

**Figure 4:**
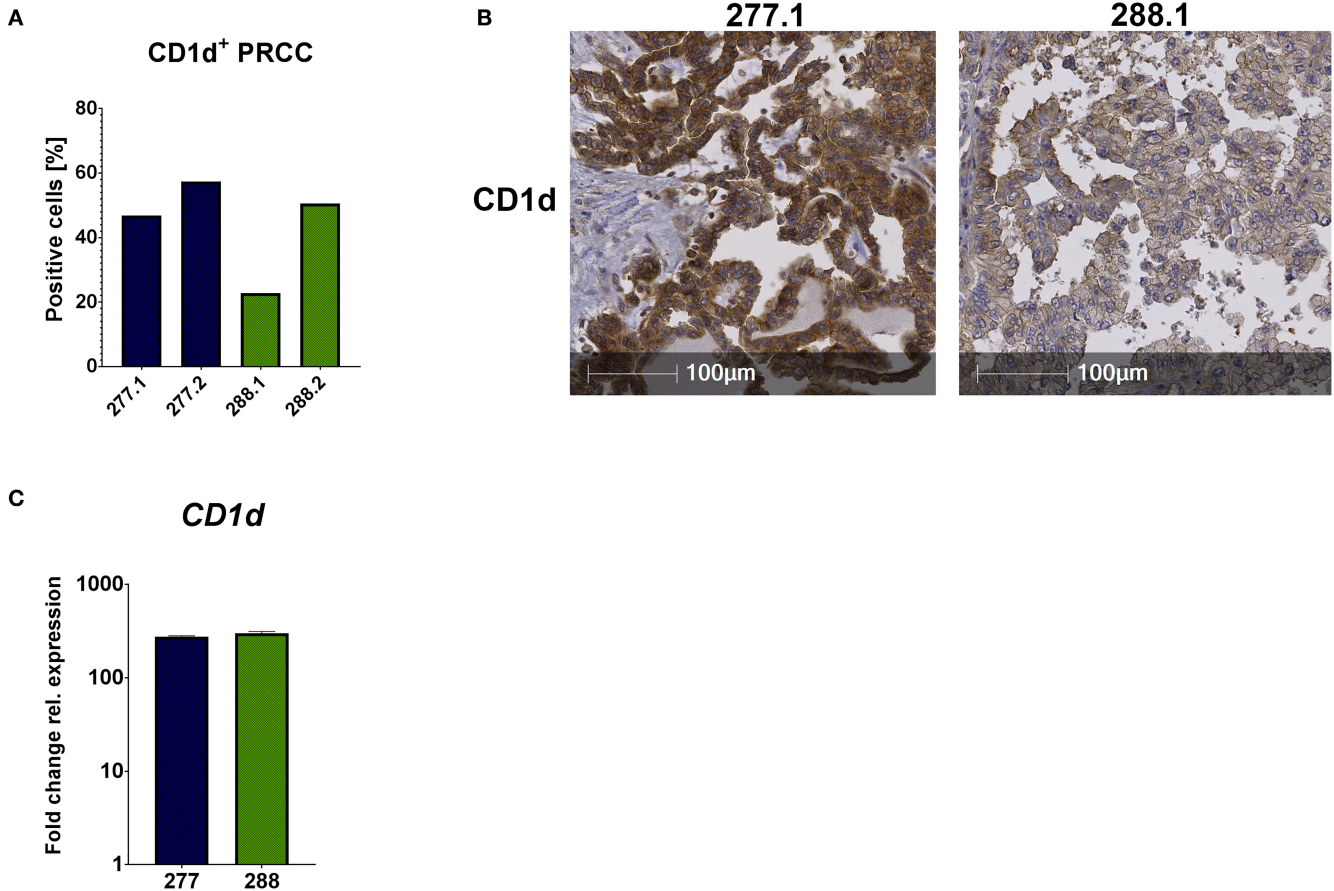
CD1d is expressed by PRCC. PRCC samples were analyzed for their CD1d expression using IHC staining and qRT-PCR. **(A)** Quantification of CD1d^+^ tumor cells of sample 277 and 288, showing high expressions of CD1d protein. Of each case two different sections were stained and analyzed (277.1, 277.2, 288.1 and 288.2). After defining the tumor area, quantification of CD1d^+^ tumor cells was performed with HALO software. **(B)** Representative immunohistochemical anti-CD1d staining of sample 277.1 and 288.1. Sample 277.1 shows a stronger CD1d expression than sample 288.1. **(C)** qRT-PCR expression analysis of *CD1d* mRNA was performed. After normalization to the housekeeping gene *HPRT*, fold change quantification was performed with respect to pediatric normal kidney tissue (sample 91). Analysis was performed in triplicates.

Unconventional T cells recognize lipid antigens presented by CD1d molecules, which have a similar structure to MHC class I complexes, but antigen recognition is independent from HLA-haplotype. In addition to the analysis of infiltrating lymphocytes, it was important to analyze the tumor samples regarding their CD1d expression for potential antigen presentation. Therefore, we performed IHC analysis and qRT-PCR. IHC analysis of FFPE samples revealed that 52.13% (mean value) of all tumor cells expressed CD1d in sample 277 and 36.67% in sample 288 (Figures 4A,B). The comparison of the CD1d and CD45 stainings revealed a moderate CD1d expression of intratumoral CD45-positive cells (Supplemental Figure 3). Furthermore, qRT-PCR analysis revealed much higher expression of *CD1d* mRNA in both PRCC samples relative to normal kidney tissue (sample 91) (Figure 4C). Overexpression of CD1d compared to normal kidney tissue has already been reported in the literature (35). In conclusion, pediatric PRCC samples displayed high expressions of CD1d.

### Tumor Own Lipid Fractions Induce the Proliferation of Unconventional T Cells From Healthy Donors and Patient-Derived PBMCs

**Figure 5:**
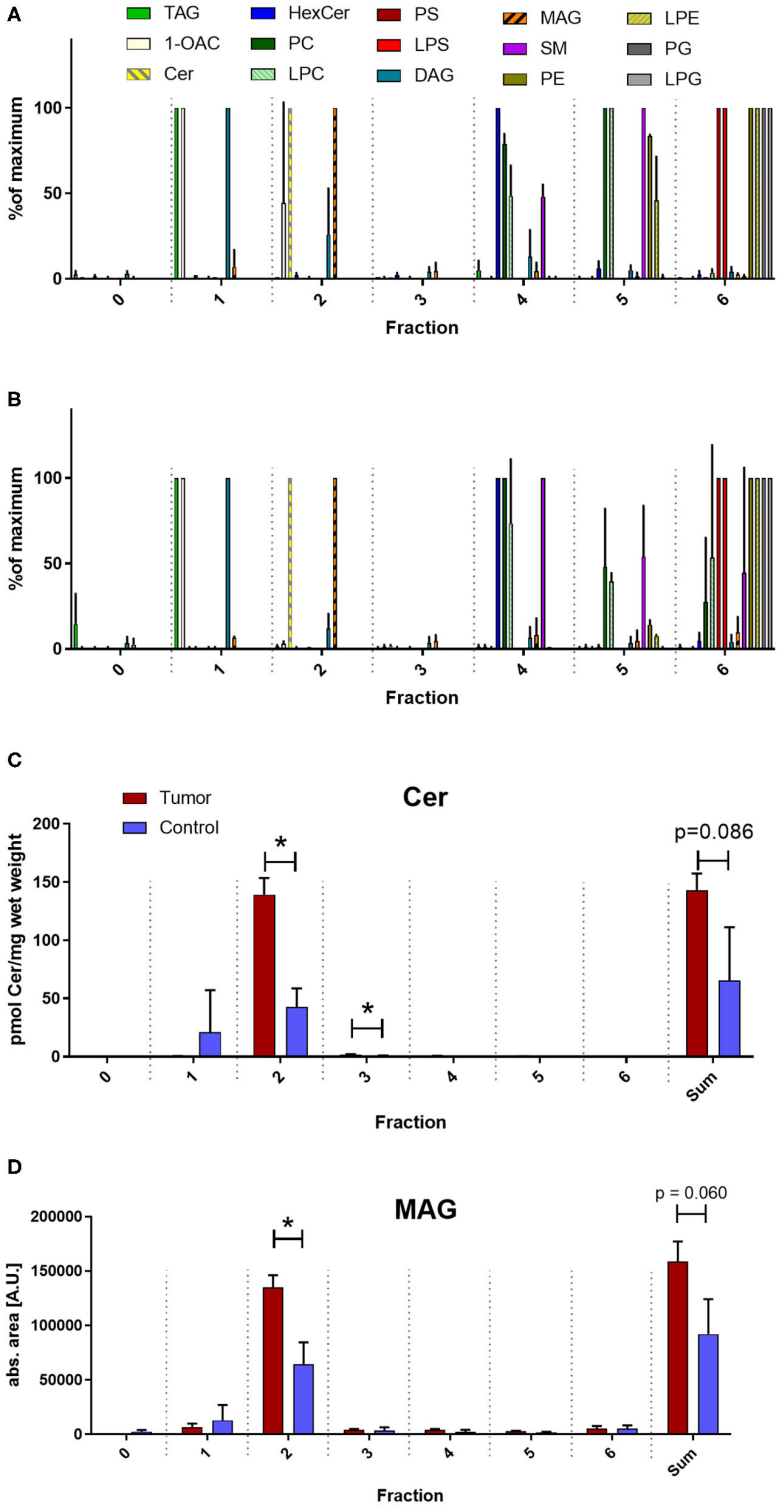
Lipid profile of fractionated tumor lipids. Lipids of tumor samples 277 **(A)** and 288 **(B)** and three normal kidneys (Supplemental Figure 5) were fractionated on aminoproyl column as described in the methods section and fractions were screened for several lipid classes by reversed phase LC-MS^2^. Note, that from all investigated lipids only ceramides (Cer) and monoacylglycerides (MAG) peak in fraction 2. Cer were quantified absolutely using internal standard **(C)** and MAG were quantified relatively using the peak area **(D)** and the level of expression between the average of the two tumor samples (red) and of the three normal kidneys (blue) was compared using student's *t*-test. Sum indicates the amount of the indicated lipid over all fractions. An asterisk denotes a significant p value:*p < 0.05). TAG, triacylglycerides; 1OAC, 1-O-acylceramides; HexCer, hexosylceramides (glucosylceramides and galactosylceramides); PC, phosphatidylcholines; LPC, lyso-PC; PS, phosphatidylserines; LPS, lysopho-PS; DAG, diacylglycerides; SM, sphingomyelines; PE, phosphatidylethanolamines; LPE, lyso-PE; PG, phosphatidylglycerols; LPG, lyso-PG. In **(A)** and **(B)** lipids were normalized to the fraction with the corresponding maximal concentration.

**Figure 6:**
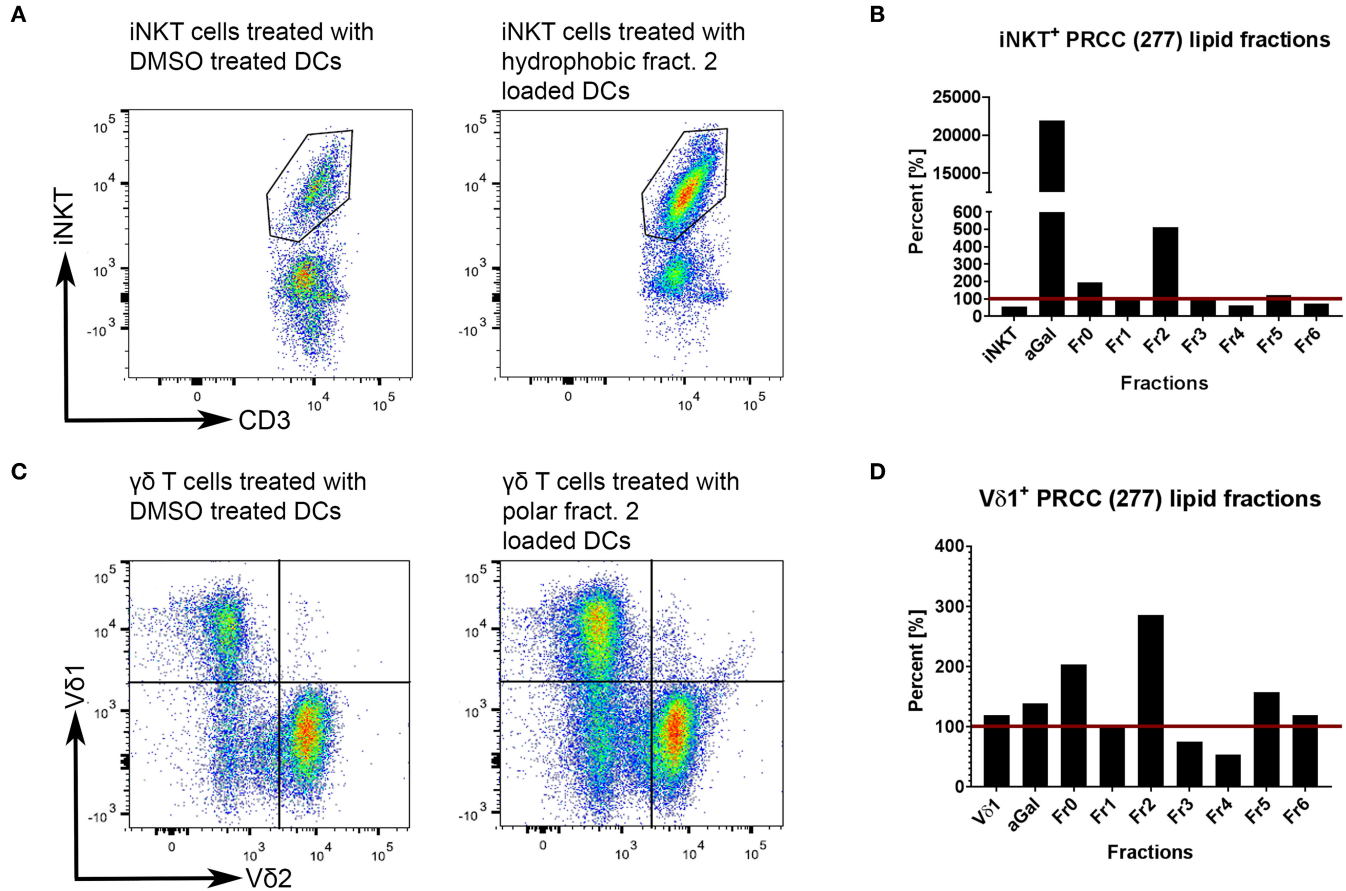
Tumor own lipids (277) induce proliferation of unconventional T cells from healthy donors. Lipid-loaded DCs were co-cultivated with purified iNKT **(A,B)** or γδ T cells **(C,D)**. DMSO was used as negative control, α-GalCer as positive control. Untreated iNKT and Vδ1 cells are shown to assess whether DMSO-treated immature DCs have an effect on the respective T cell population. **(A,B)** After three stimulations with lipid-loaded DCs, lipid fraction 2 showed the strongest induction of proliferation of healthy donor 2-derived iNKT cells compared to DMSO control (defined as 100%, red line). DMSO has a small stimulating effect on iNKT cells. **(C,D)** After three stimulations, lipid fraction 2 is able to induce the proliferation of healthy donor 4-derived Vδ1^+^ T cells compared to DMSO control (defined as 100%, red line). DMSO has no stimulating effect on Vδ1^+^ T cells. The results for all lipid fractions and all donors are shown in Supplemental Figure 8.

Unconventional T cells are known to bind lipid antigens and therefore we fractionated tumor lipids of PRCC samples 277 and 288 in seven fractions to test if individual fractions would affect these T cells. In both samples, early fractions contained more hydrophobic lipids, i.e., triacylglycerides, 1-O-acylceramides and diacylglycerides peaked in fraction 1 and ceramides and monoacylglycerides concentrated in fraction 2. Polar lipids eluted with later fractions: Hexosylceramides (including glucosyl- and galactosylceramides) eluted with fraction 4, whereas most of the phosphatidylcholine, sphingomyeline and lyso-phosphatidylcholine were present in fraction 5 and anionic lipids eluted in fraction 6 (Figures 5A,B). Fractionation of lipids of three normal pediatric kidneys showed a similar distribution profile (Supplemental Figure 4), but the concentration of ceramides and monoacylglycerides was significantly increased in the tumor samples in fraction 2 (Figures 5C,D), while no difference was observed in the amount of other lipids (Supplemental Figures 5, 6). The lipid fractions of sample 277 were analyzed by an *in vitro* assay to see if they were able to induce the proliferation of unconventional T cells. Therefore, lipid fractions were loaded on CD1d-positive, immature dendritic cells. Subsequently, lipid-loaded DCs were co-cultivated with iNKT or γδ T cells and the proliferation of iNKT and γδ T cells was measured by flow cytometry. The immunogenic potential of lipid fractions was tested on unconventional T cells from six healthy donors. Considering the variability between the different donors, fractions were considered immunogenic if they increased cell population size by more than 30% relative to the DMSO vehicle control. α-GalCer was used as positive control. All results are listed in Table 1, revealing that several fractions are able to induce the proliferation of unconventional T cells. In some samples, the use of DMSO had a small effect on iNKT or γδ T cells, but this effect was much smaller than the effect of the lipid fractions. Particularly, compared to DMSO control, fraction 2 showed a strong potential to stimulate the proliferation of iNKT cells (Figures 6A,B) in three healthy donors, whereas the proliferation of Vδ1 T cells from different donors was not induced by a common but by individual lipid fractions (Figures 6C,D). Similar results were obtained when fractions 2 and 5 isolated from the tumor sample 288 were used to stimulate unconventional T cells (Supplemental Figure 7).

**Table 1:** Lipid fractions of pediatric PRCC are able to induce proliferation of unconventional T cells.

**Buffy coats**	**Population size of iNKT cells relative to DMSO**		**Population size of V****δ****1 cells relative to DMSO**	
	****α**-GalCer**	**Lipid fraction**	****α**-GalCer**	**Lipid fraction**
Healthy donor 1	124%	Fraction 5 (233%)	106%	Fraction 6 (135%)
Healthy donor 2	21,951%	Fraction 0 (196%) Fraction 2 (511%)	120%	Fraction 0 (141%)
Healthy donor 3	178%	No positive fraction	118%	Fraction 2 (131%) Fraction 3 (222%) Fraction 5 (148%)
Healthy donor 4	215%	Fraction 3 (187%)	139%	Fraction 0 (204%) Fraction 2 (286%) Fraction 5 (158%)
Healthy donor 5	6,285%	Fraction 2 (1,677%) Fraction 3 (259%)	49%	No positive fraction
Healthy donor 6	11,196%	Fraction 2 (173%)	121%	Fraction 1 (256%)

*Increase in T cell populations following stimulation with α-GalCer or lipid fractions relative to DMSO-treated cell populations (vehicle control). The value of the vehicle control was defined as 100%. Those fractions inducing proliferation > 30% were considered as immunogenic*.

Statistical analyses with the non-parametric Kruskal-Wallis test showed neither significant differences between DMSO and the individual fractions nor between the fractions among themselves (Supplemental Figure 8). Significant effect of α-GalCer stimulation on the amount of iNKT cells could only be detected relative to DMSO in sample 277 (^*^p = 0.0179; data not shown). High data variances even in the positive control might be due to strong differences in the physiology, constitution and cell distribution in general of the healthy donors, illustrating the strong impact of different parameters on immune cell composition. Nevertheless, there is a strong tendency for fraction 2 to stimulate unconventional T cells.

**Figure 7:**
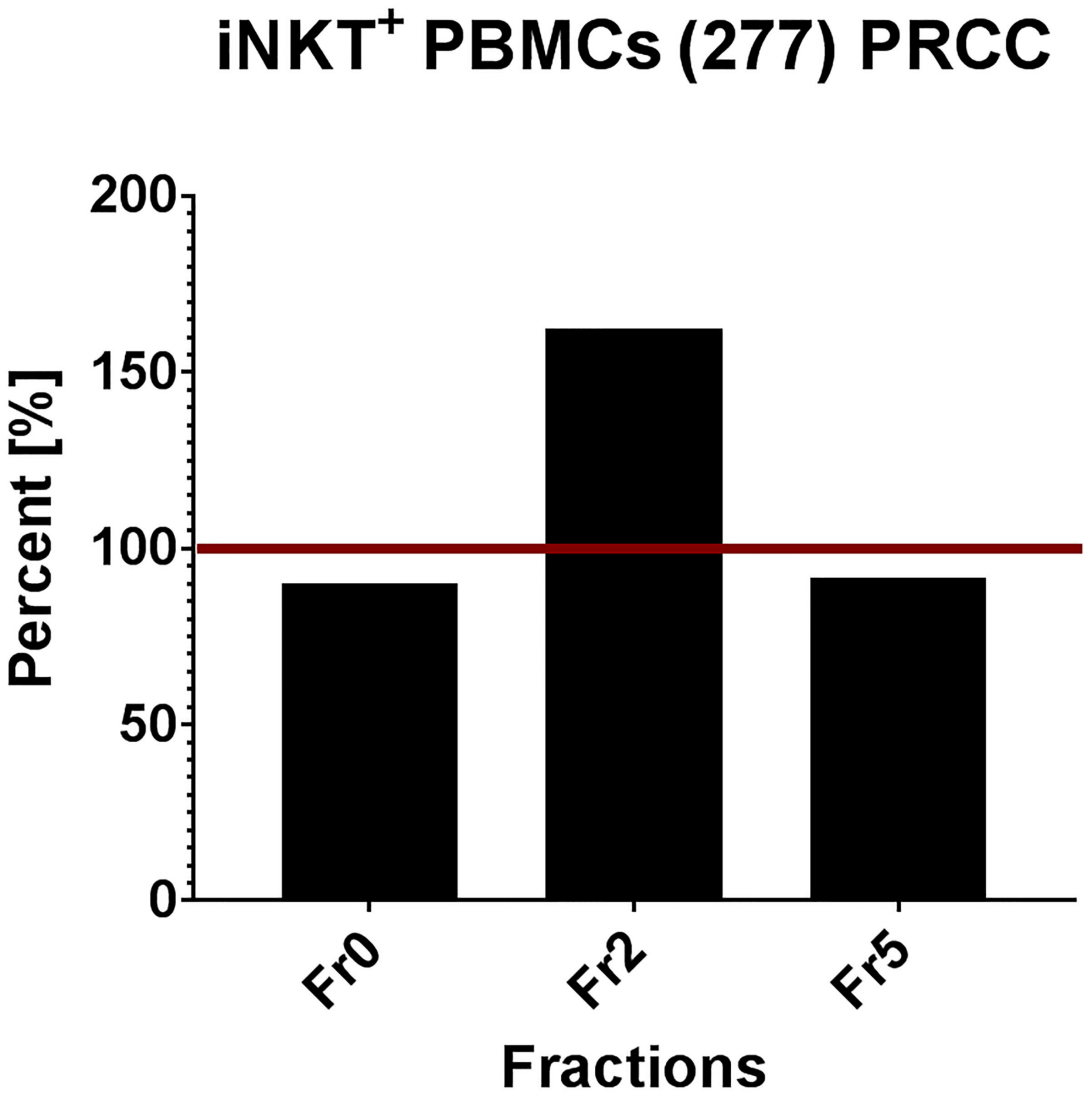
Tumor own lipids (277) induce proliferation of iNKT cells from patient-derived PBMCs (*n* = 1). Quantification of flow cytometric analysis showing the percentage of iNKT cells in a patient-derived PBMC population stimulated with lipid fractions 0, 2, and 5 relative to the positive control α-GalCer (defined as 100%, red line).

In order to validate the results obtained from T cell subsets of healthy donors, the same *in vitro* assays were performed with patient-derived (277) iNKT cells. Therefore, we pre-selected potential immunogenic lipid fractions and performed the experiment with fractions 0, 2 and 5 (Figure 7). Fraction 2 strongly induced the proliferation of iNKT cells derived from patients PBMCs. Interestingly, the induction was even stronger than with α-GalCer, which served as positive control. The remaining lipid fractions were not able to stimulate iNKT cells obtained from patients PBMCs (data not shown).

In conclusion, we identified different lipid fractions from pediatric PRCC sample 277 which induced the proliferation of unconventional T cells from healthy donors. Based on these results, we analyzed selected lipid fractions for their ability to induce proliferation of patient's own immune cells. Fraction 2, indeed, induced proliferation of iNKT cells isolated from patient's PBMCs.

### Tumor Lipids Are Presented via CD1d to iNKT Cells

**Figure 8:**
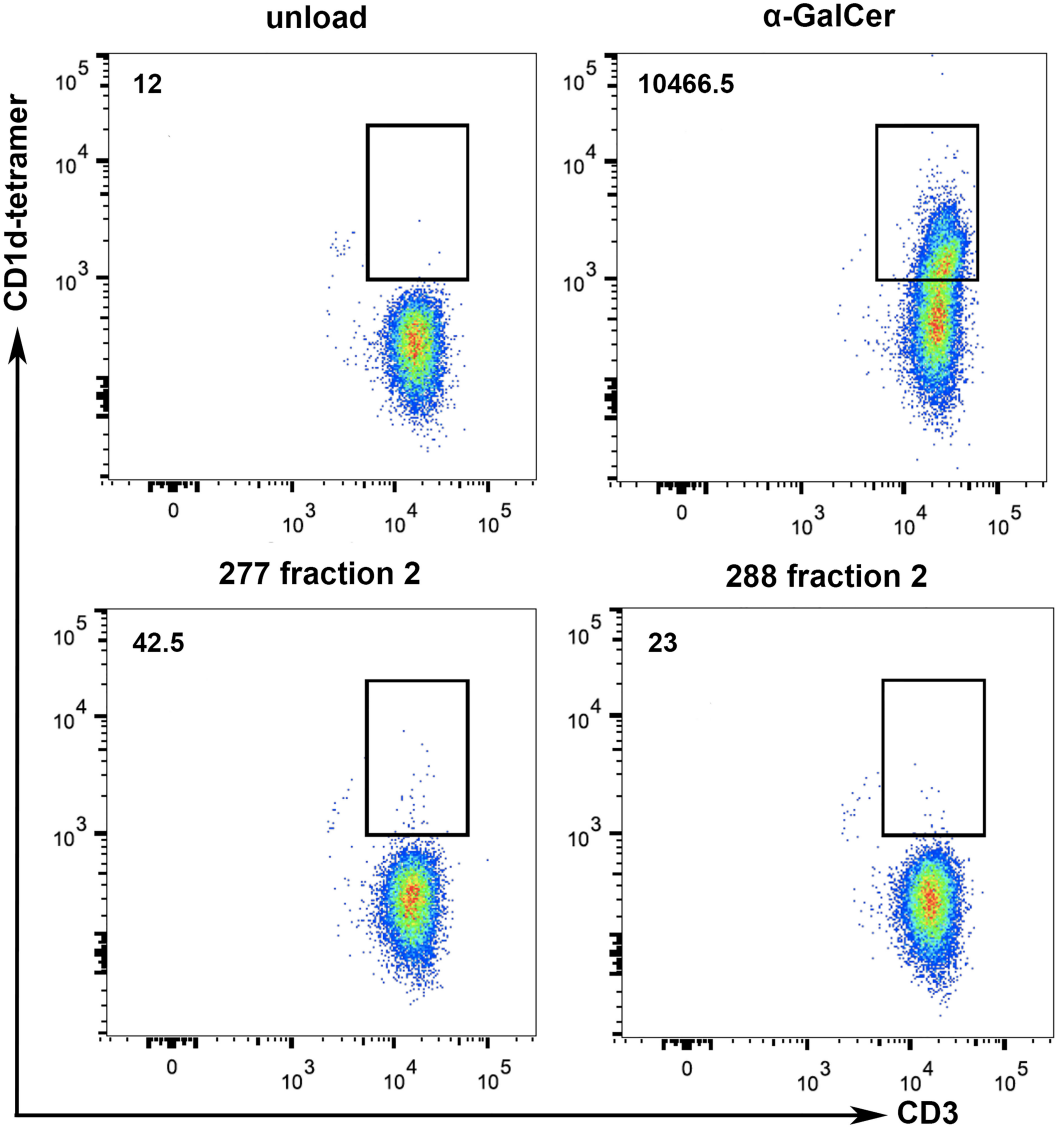
Tumor lipids are presented to iNKT cells by CD1d. To show whether the stimulation of iNKT cells is dependent on CD1d specific lipid presentation, CD1d tetramer stainings with iNKT cells of two healthy donors were performed. α-GalCer-loaded CD1d tetramers were used as positive control and lipid-unloaded, vehicle-treated CD1d tetramers as negative control. Incubation of iNKT cells with lipid fraction (277 Fr. 2 and 288 Fr. 2) loaded CD1d tetramers reveal a small CD3^+^CD1d-tetramer^+^ population, indicating CD1d-specific presentation of tumor derived lipids. Numbers on the upper left represent the mean values of total CD3^+^CD1d-tetramer^+^ cells from the two donors.

Methods for characterization of antigen recognition by iNKT cells rely on direct detection of CD1d-lipid complexes bound to the NKT cell receptor using tetramers. To further specify whether lipid fraction 2-induced proliferation of iNKT cells is dependent on CD1d presentation, we performed CD1d tetramer stainings with iNKT cells isolated from two healthy donors. The analyses revealed small CD3^+^CD1d tetramer^+^ populations due to stimulation with 277 fraction 2 and 288 fraction 2-loaded CD1d tetramers (Figure 8). Incubation of iNKT cells with α-GalCer-loaded CD1d tetramers revealed a big CD3^+^CD1d tetramer^+^ population, indicating a strong CD1d-specific presentation. It is worth noting that while α-GalCer is provided as purified lipid, we don't know the final concentration of the antigen lipid in fraction 2. Therefore, a large population as detected after incubation with α-GalCer cannot be expected. These results indicate the existence of a subset of iNKT that can bind a lipid present in fraction 2 presented via CD1d.

## Discussion

In contrast to adult tumor entities, little is known about tumor-infiltrating lymphocytes in pediatric, solid tumor entities. Most pediatric studies in context of TILs focused on the infiltration of conventional T cells (15, 17–19, 36), whereas infiltration of γδ T cells is described in pediatric medulloblastoma (14) and of iNKT cells is described in neuroblastoma (16, 31). To date and to our knowledge, there is no data available that investigates infiltrating lymphocytes in pediatric PRCC. The data of the present study suggest an infiltration of both conventional and unconventional T cells in the tumor of pediatric PRCC. As tumor material of this entity is very rare, our analysis is based on two pediatric PRCC samples. T cell infiltration was detected by flow cytometry and, particularly, infiltrations of unconventional T cells were validated by immunohistochemistry and qRT-PCR. Infiltrating γδ T cells are already described in adult renal cell carcinoma (37). In the present work, we also identified infiltrating γδ T cells in pediatric PRCC. Compared to the T cell frequency in patients peripheral blood cells, there is an increased amount of iNKT cells (sample 277) and γδ T cells (sample 288) found in the tumor. Infiltrating γδ T cells and iNKT cells contribute to natural tumor immunosurveillance. Anti-cancer functions of γδ T cells are due to the interaction with other immune cells or due to direct lysis of tumor cells e.g., via the perforin-granzyme pathway (22). iNKT cells have a potent anti-tumor activity by bridging innate and adaptive immune system. Once activated, iNKT cells produce various cytokines that activate several other anti-tumor immune cells (38). Moreover, iNKT cells have the ability to kill CD1d-expressing tumor cells directly via the perforin/granzyme B, Fas-Fas ligand system and tumor necrosis factor-α-related apoptosis-inducing ligand (TRAIL) (23). Another interesting ability of activated iNKT cells is the reinvigoration of exhausted immune cells in the tumor microenvironment (38). Therefore, the infiltration of unconventional T cells in pediatric PRCC identified in the present work suggests these cells as important effectors in antitumor immunity. Nonetheless, further pediatric PRCCs should be screened for their infiltration by unconventional T cells.

The tumor immunogenicity is largely dependent on the composition of the tumor microenvironment and, thus, defines conditions for a potential lymphocyte infiltration and antigen presentation. Beside the production of chemokines, the existence of antigen-presenting structures within the tumor is crucial for activation of T cell responses (39). CD1d is a MHC class I-like molecule and serves as an antigen-presenting structure for iNKT and Vδ1^+^ γδ T cells (24, 40). The advantage of CD1d-expressing tumors is the independence of MHC molecules (41). Several vaccine and T cell therapies are based on immunogenic, mutated peptides resulting from genetic alterations within the tumor. These peptides are presented on MHC molecules of cancer cells (42). In contrast to adult tumors, pediatric tumors are usually associated with low mutation burden. This is why MHC peptide-based immunotherapy is less efficient in pediatric cancer patients (43). Among pediatric tumors, increased CD1d expression was already detected in medulloblastoma with SHH subtype, thus suggesting an immunotherapeutic option with iNKT cells (44). Moreover, a murine study with breast cancer cells showed that a downregulation of CD1d results in inhibition of iNKT-related antitumor immunity and promotion of metastasis (45). In adult RCCs, a high expression of CD1d is associated with poorer prognosis (35). However, high expression of CD1d is not always associated with iNKT infiltration, as shown by Metelitsa and colleagues in neuroblastoma samples (31). To date, there is no information about CD1d expression in pediatric PRCC. Our data suggest a strong expression of CD1d, especially in sample 277. In both cases, surgery was the treatment of choice without any adjuvant therapy and both patients did not experience a recurrence of their disease during the last 2 years. These facts would not suggest poor prognosis of CD1d expression in case of pediatric PRCCs.

The recognition of lipids, as well as of peptides, allows the immune system to detect a wide range of antigenic molecules. Lipid-specific responses play a role in both defense against infection and in autoimmunity, as well as in the immunological control of tumors. Thus, it is important to understand the mechanisms of T cell lipid recognition (46). Lipid antigens are presented via CD1 molecules that have a hydrophobic binding groove. This allows the binding of hydrophobic lipid antigens without additional processing, which is why a higher efficiency of hydrophobic antigens is to be assumed. Furthermore, it has been recognized that the length of the hydrophobic chain has a great impact and that the length of lipids bound to human CD1d molecules modulates the affinity of NKT cell TCR and the threshold of iNKT cell activation (25, 47). In contrast, polar lipids contain hydrophilic groups. It is known that e.g., hydrophilic glycolipids require additional processing of the sugar residues by lysosomal hydrolases (48). Thus, the endogenous gangliosides GM3 and GD3 need to be processed via the lysosome in order to activate iNKT cells via CD1d (49). Our data suggest a stimulation of iNKT cells by the fraction 2 of pediatric PRCC sample 277 and 288, containing relative hydrophobic membrane lipids and lysolipids. The stimulation was detected in cells of healthy donors and in the patient's own cells. This fraction contains ceramides and monoacylglycerols amongst others. Interestingly, the expression of ceramides and monoacylglycerols appeared to be significantly increased in the two tumor samples analyzed in this work as compared to three normal kidney samples, whereas the concentration of other lipid fractions did not differ. Ceramides are a subset of sphingolipids and amides of fatty acids with long-chain di- or tri-hydroxyl 2-amino bases. The acyl group of ceramides varies in length, saturation and hydroxylation. The nature of the base and the structure of the fatty acid thereby determine the distribution of glycolipids of the membrane within the cell, their association with other molecules, frequency of degradation and persistence within the cell (50). All of these aspects are relevant to immune recognition because T cell receptors recognize specific structures of the lipid antigens. For example, in the recognition of ganglioside GM1, the structure of the ceramide tail contributes to the specific recognition by the T cell receptor (51). Another important aspect is that different cell types and tissues synthesize and accumulate glycosphingolipids with modified ceramide structure. Modified lipid structures may arise from differences in lipid metabolism during cell growth, viral transformation or during oncogenesis and have an effect on the specific immune response (46). Moreover, sphingolipids with very-long acyl chains appear to be critical for proper iNKT cell numbers, survival and maturation in mice (52). Although ceramides, as present in fraction 2, are not known to stimulate iNKT cells via CD1d by themselves, they may be processed within the APCs to more immunogenic sphingolipids and it may be noted that at least 70% of the ceramides in fraction 2 contain very-long chain fatty acids (data not shown). Finally, immunomodulatory properties of ceramides may also depend on their concentration, as shown for β-GalCer with long acyl chains (C ≥ 12) that, when used at high concentrations, can effectively activate cytokine secretion by NKT cells (53).

The existence of a lipid with activating potential in fraction 2 was confirmed by tetramer staining, which allowed the identification and enumeration of specific CD1d-restricted iNKT cells. The determination of the exact structure of this lipid will require further sub-fractionation, activity testing and a different type of sophisticated MS analysis (untargeted analysis with high resolution mass spectrometry). Moreover, the effect of the lipid ligand on the cytokine profile and therefore on the immunomodulatory potential of iNKT cells remain to be clarified.

In the context of γδ T cells, CD1d is known to present lipid antigens for Vδ1 and Vδ3-positive cells (54, 55). Several studies about activation of Vδ1-positive γδ T cells used lipids such as sulfatides and α-galactosylceramide. Analyses of different TCR clones showed a specificity for sulfatides but also the ability to recognize other endogenous lipids presented via CD1d. Moreover, one clone was specific for the CD1d-α-GalCer complex (56). Other studies have shown that Vδ1-positive T cells have TCR structures similar to those of type II NKT cells and recognize CD1d-presented sulfatides and lysosulfatides (57, 58). In our project, sulfatides are to be assumed in fraction 6. A significant stimulation by this fraction was not observed in our *in vitro* studies. Other lipid fractions rather induced the proliferation of Vδ1^+^ T cells in a donor-dependent manner.

Previous clinical studies reported on injection of α-GalCer-loaded immature DCs or mature monocyte-derived DCs in patients with multiple myeloma and other solid tumors, which led to a reduction of tumor markers or tumor mass or to stable metastatic disease (59–61). Furthermore, Lepore et al. were able to identify own lipids of leukemic blasts presented via CD1c, which are recognized by T cells and cause the specific elimination of the blasts (62). Thus, lipid antigens might have high potential for novel therapeutic approaches.

In conclusion, we demonstrate the presence of tumor-infiltrating unconventional T cells in two pediatric PRCC samples. Furthermore, a strong expression of CD1d molecules by tumor cells was observed in both samples, which function as a lipid antigen-presenting structure. Indeed, tumor own lipid fractions of pediatric PRCC were able to induce the proliferation of unconventional T cell subsets and may offer new therapeutic strategies for pediatric PRCC.

## Data Availability Statement

The datasets generated for this study are available on request to the corresponding author.

## Ethics Statement

Ethical review and approval was not required for the study on human participants in accordance with the local legislation and institutional requirements. Written informed consent to participate in this study was provided by the participants' legal guardian/next of kin.

## Author's Note

This publication contains data from the dissertation of NL.

## Author Contributions

CP and JF initiated the project. NL planned, NL and NZ performed the experiments. NL, CP, NZ, KE, AR, MN, and AW interpreted data. LS performed and interpreted IHC analyses. SF performed quantification analyses on IHC stainings. LR and NB provided technical support. RS performed lipid fractionation protocols and the analysis of lipid classes. The project was supervised by CP and JF. NL and CP wrote the manuscript. All authors reviewed and edited the manuscript.

## Conflict of Interest

This publication contains data from the dissertation of NL. The remaining authors declare that the research was conducted in the absence of any commercial or financial relationships that could be construed as a potential conflict of interest.
