# Supplementary material for: Tumor Lipids of Pediatric Papillary Renal Cell Carcinoma Stimulate Unconventional T Cells
Source: Front Immunol. 2020 Aug 20;11:1819. doi: 10.3389/fimmu.2020.01819 (PMC7468390; doi:10.3389/fimmu.2020.01819)
Supplement: Supplementary file 1 [file Data_Sheet_1.docx]

**Supplemental Figure 1**

**
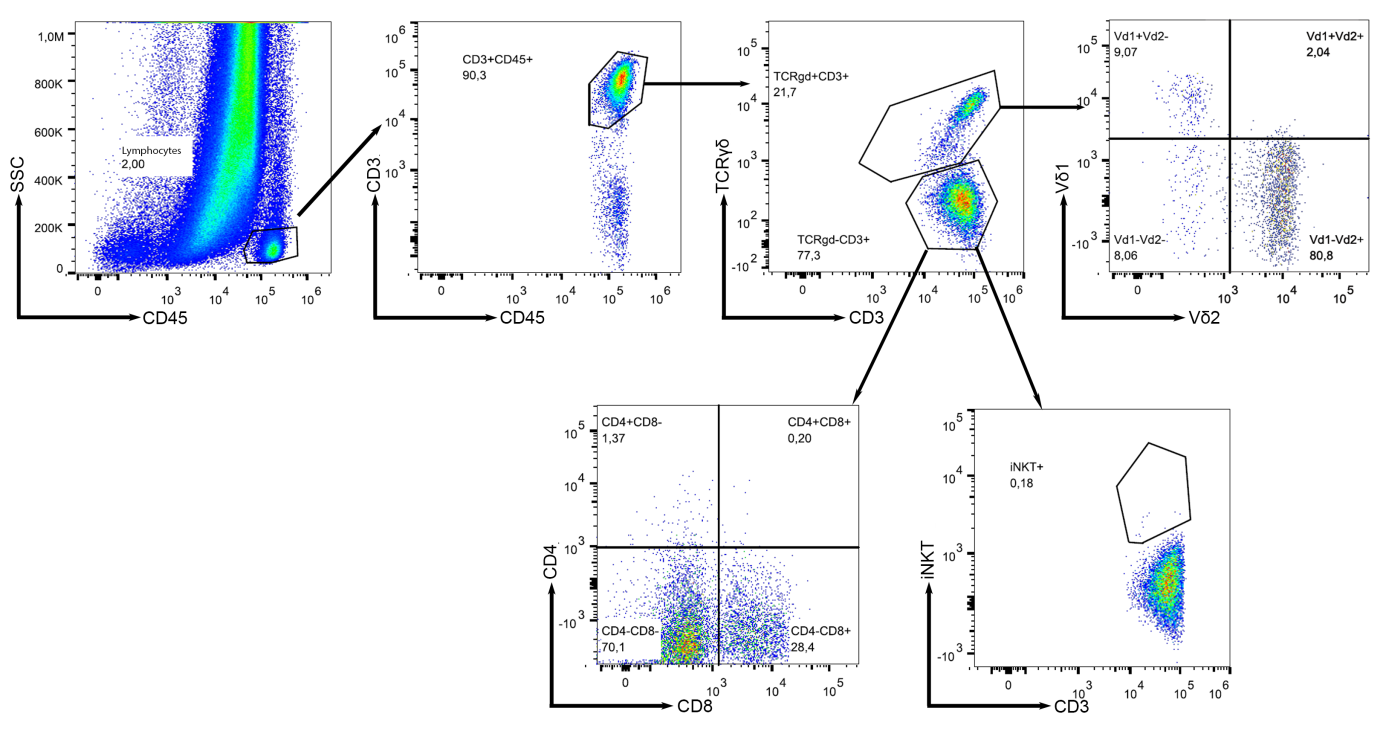
**

**Supplemental Figure 1: Gating strategy for characterization of TILs (sample 288)**

First, lymphocyte population was defined in SSC CD45 gate. After gating the CD3^+^CD45^+^ population, TCRgd^+^CD3^+^ and TCRgd^-^CD3^+^ populations were specified. TCRgd^+^CD3^+^ population was used to define γδ T cell subsets Vδ1 and Vδ2. TCRgd^-^CD3^+^ population was utilized for characterization of CD4^+^ and CD8^+^ T cells as well as for characterization of iNKT cells (iNKT^+^CD3^+^).

**Supplemental Figure 2**

**
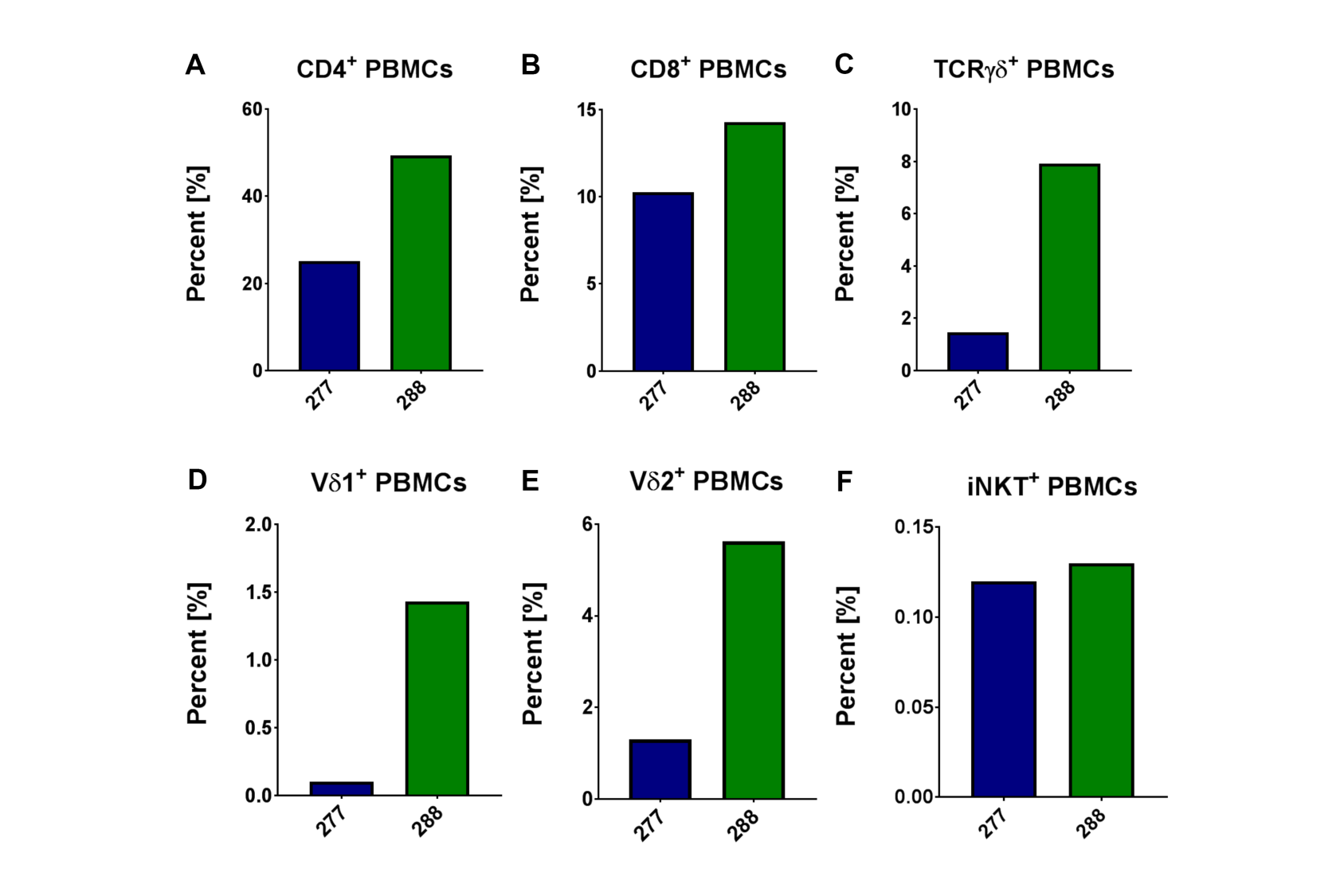
**

**Supplemental Figure 2: Flow cytometric distribution of PBMCs in pediatric PRCC patients (277 and 288)**

Flow cytometric analysis was performed using specific antibodies against CD4, CD8, TCRγδ, Vδ1, Vδ2 and iNKT T cell receptor. The distribution of different subpopulations within the lymphocyte population is shown. **(A)** CD4^+^ T cells; **(B)** CD8^+^ T cells; **(C)** TCRgd^+^ T cells; **(D)** Vd1^+^ T cells; **(E)** Vd2^+^ T cells, **(F)** iNKT cells.

**Supplemental Figure 3**

**
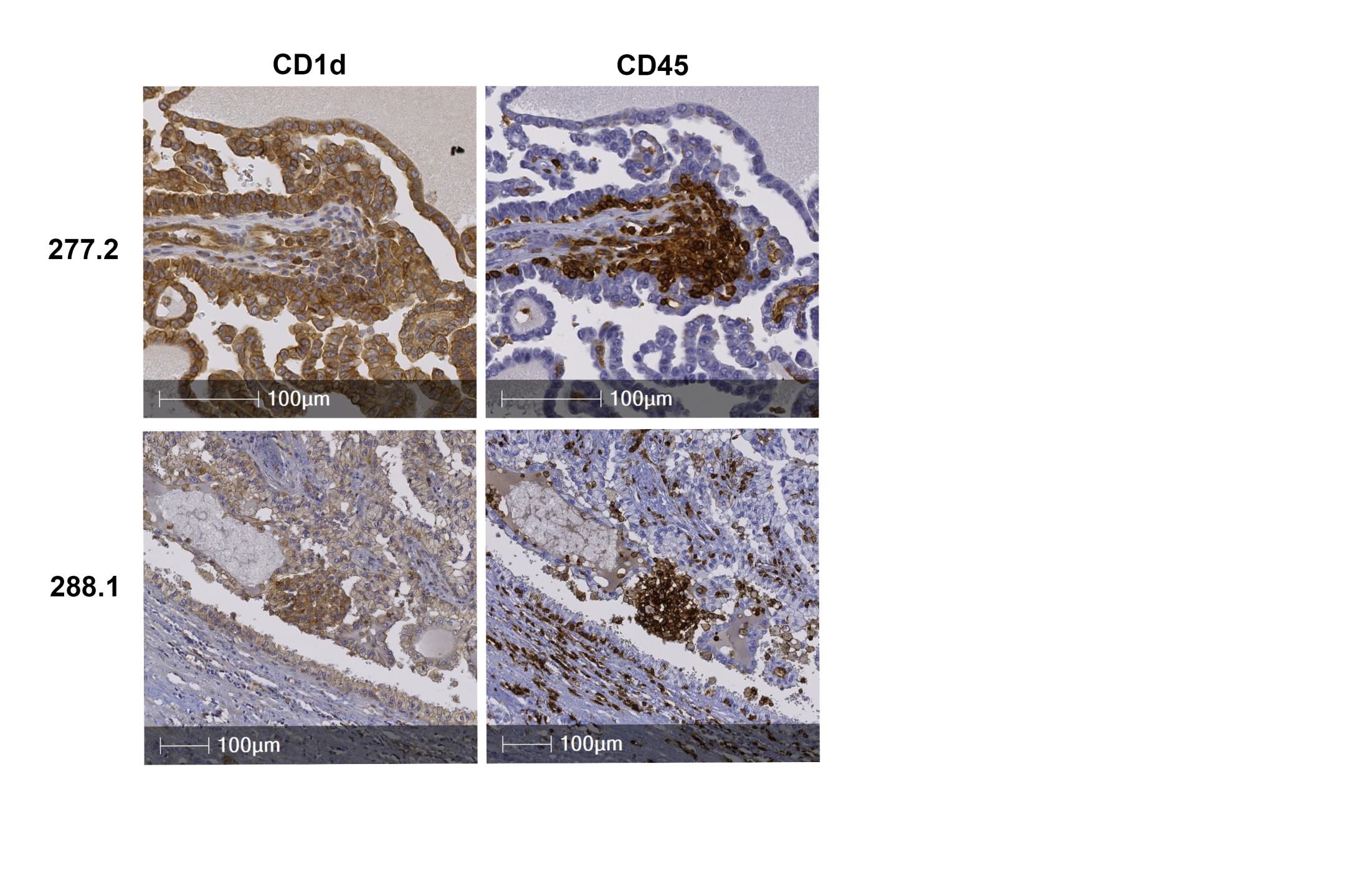
**

**Supplemental Figure 3: CD1d expressing CD45^+^ intratumoral cells**

Comparison of CD1d and CD45 immunohistochemical stainings of FFPE samples 277.2 and 288.1. Intratumoral CD45^+^ cells express moderate amounts of CD1d.

**Supplemental Figure 4**

**
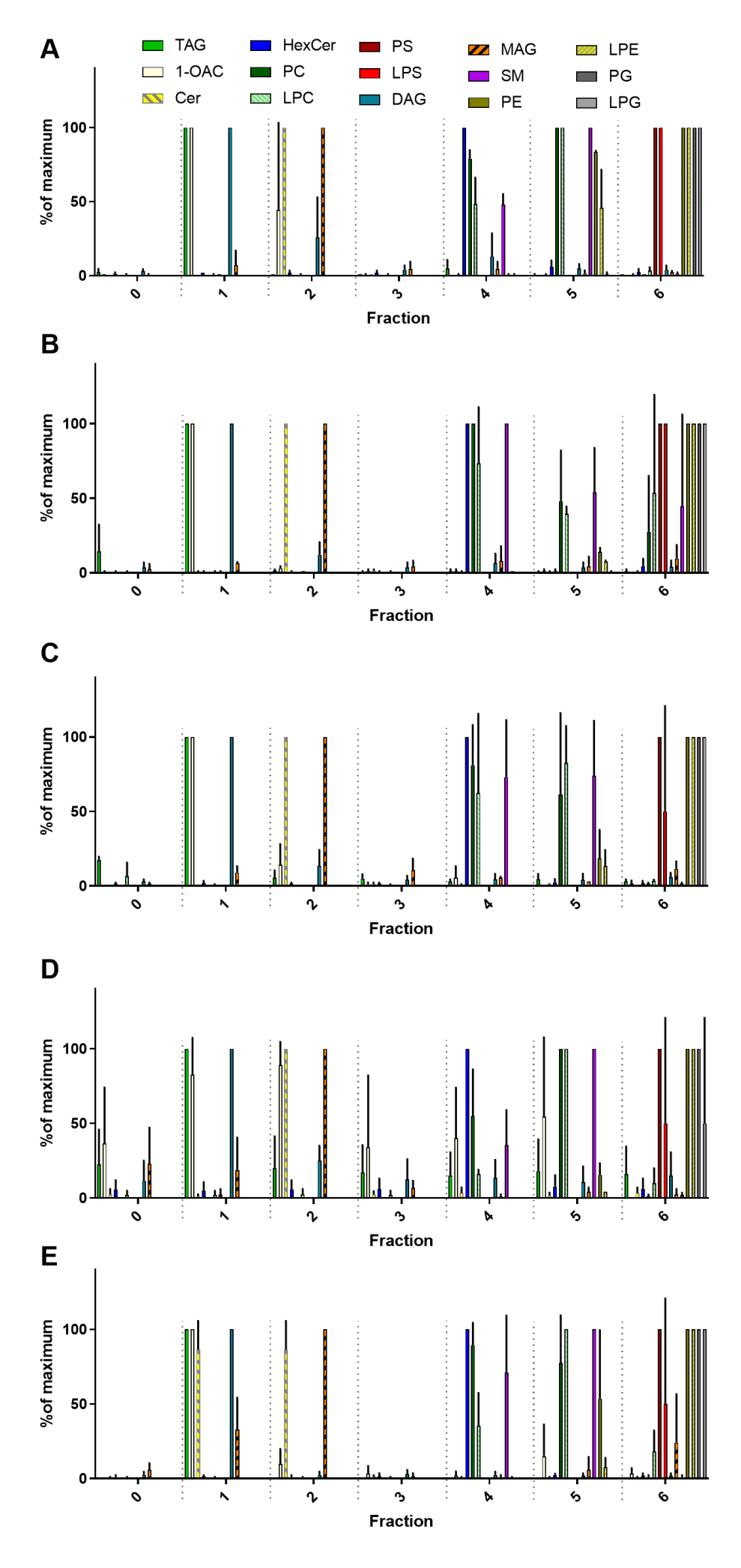
**

**Supplemental figure 4: Lipid profile of fractionated lipids extracted from PRCC samples and normal kidney samples**

Lipids of tumor samples no. 277 **(A)** and no. 288 **(B)** and of normal kidneys no. 98 **(C)**, no. 181 **(D)** and no. 206 **(E)** were fractionated on aminoproyl columns as described in the methods section and fractions were screened for several lipid classes by reversed phase LC-MS^2^. TAG, triacylglycerides; 1OAC, 1-O-acylceramides; HexCer, hexosylceramides (likely glucosylceramides); PC, phosphatidylcholines; LPC, lyso-PC; PS, phosphatidylserines; LPS, lysopho-PS; DAG, diacylglycerides; SM, sphingomyelines; PE, phosphatidylethanolamines; LPE, lyso-PE; PG, phosphatidylglycerols; LPG, lyso-PG, ceramides (Cer), monoacylglycerides (MAG). Lipids were normalized to the fraction with the corresponding maximal concentration.

**Supplemental Figure 5**

**
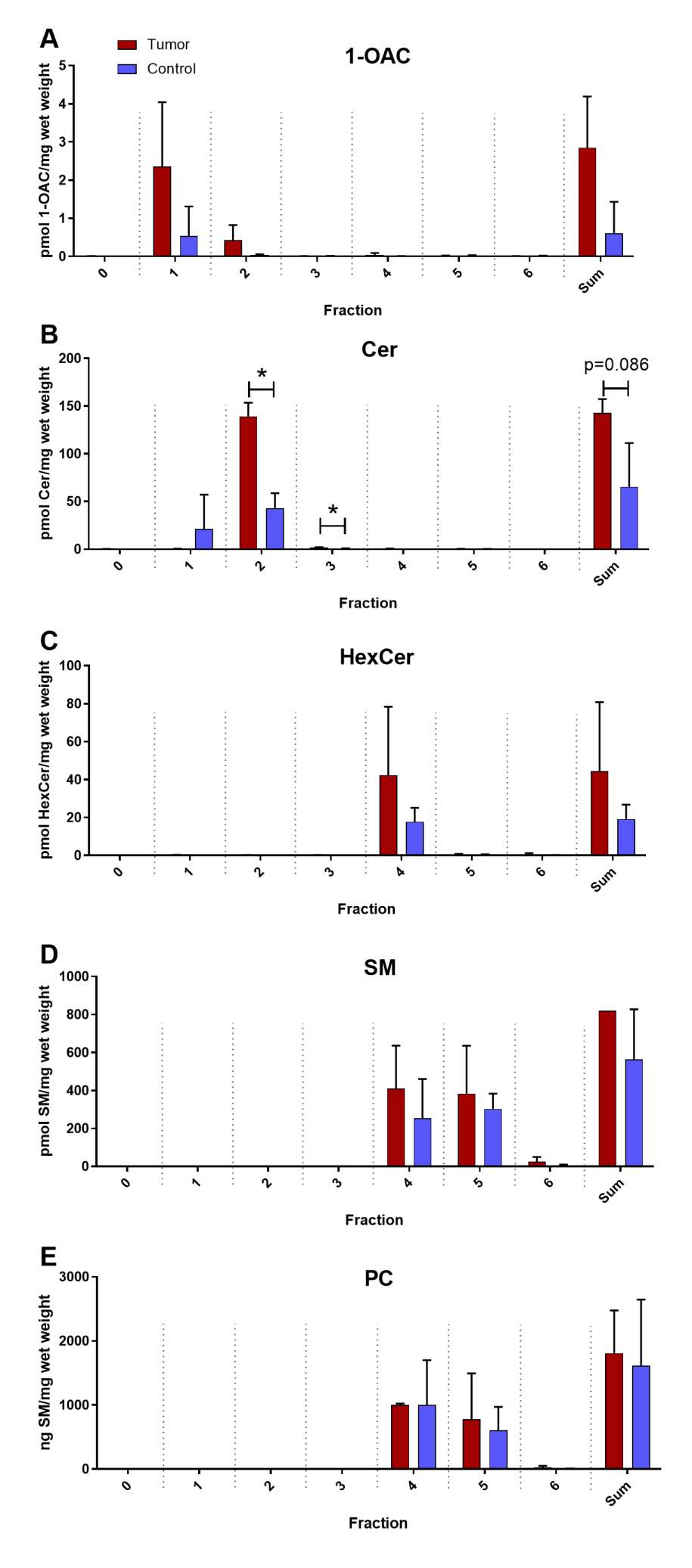
**

**Supplemental figure 5: Quantification of selected lipids in PRCC samples and normal kidney samples using internal standards**

Lipids of tumor samples no. 277 and no. 288 and of normal kidneys no. 98, no. 181 and no. 206 were fractionated on aminoproyl columns as described in the methods section and fractions were screened for several lipid classes by reversed phase LC-MS^2^. The indicated lipids were quantified using internal standards and the average amount of lipids of the two tumor samples (red) and of the three normal kidneys (blue) was compared using student’s t-test. Sum indicates the amount of the indicated lipid in all fractions. An asterisk denotes a significant p value: * p<0.05. 1OAC, 1-O-acylceramides; Cer, ceramides; HexCer, hexosylceramides (glucosylceramides and galactosylceramides); SM, sphingomyelines; PC, phosphatidylcholines.

**Supplemental Figure 6**

**
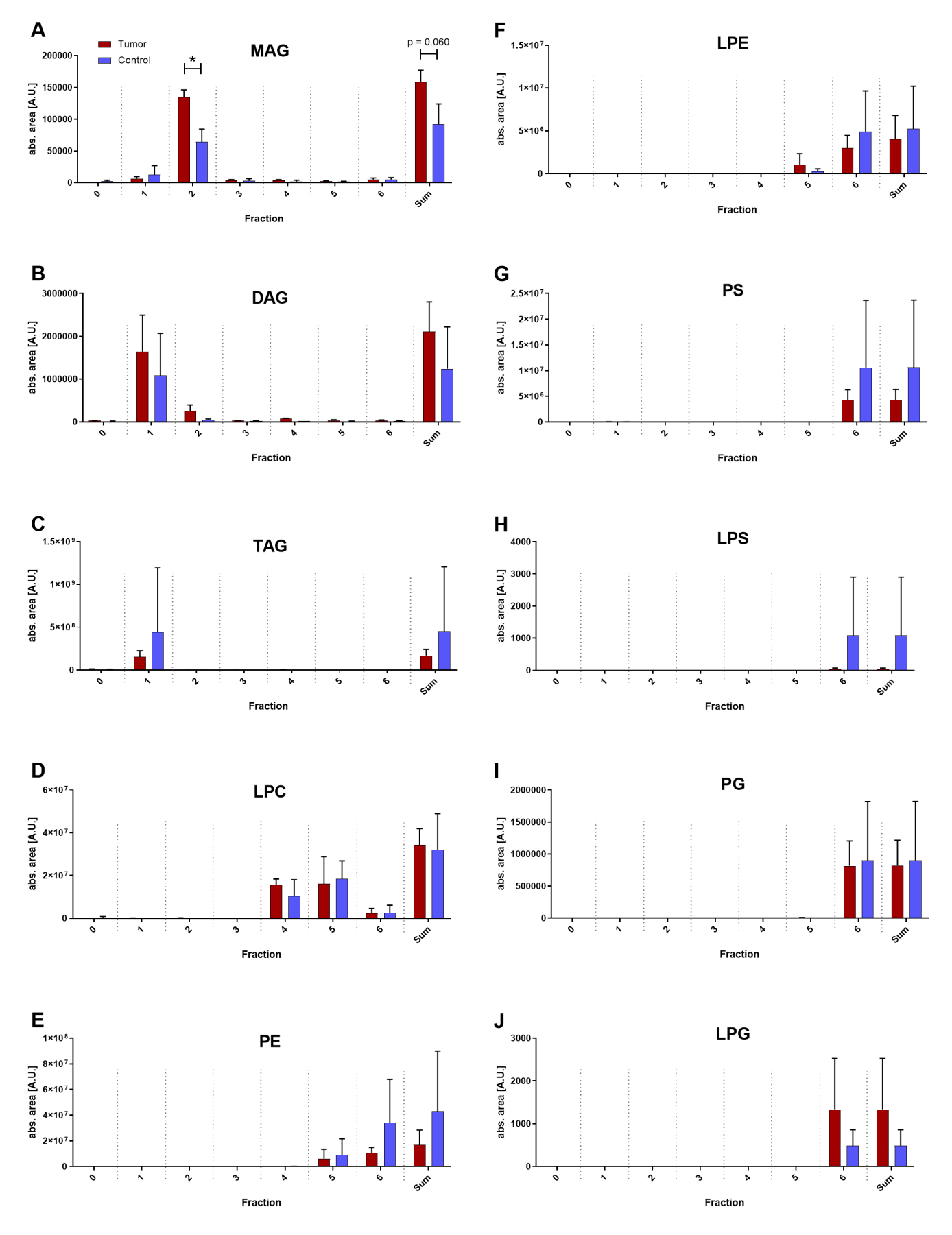
**

**Supplemental figure 6: Quantification of selected lipids in PRCC samples and normal kidney samples**

Lipids of tumor samples no. 277 and no. 288 and of normal kidneys no. 98, no. 181 and no. 206 were fractionated on aminoproyl columns as described in the methods section and fractions were screened for several lipid classes by reversed phase LC-MS^2^. Lipids were quantified relatively using the area of the peaks and the level of expression between the average of the two tumor samples (red) and of the three normal kidneys (blue) was compared using Student’s t-test. Sum indicates the amount of the indicated lipid over all fractions. TAG, triacylglycerides; LPC, lyso-PC; PS, phosphatidylserines; LPS, lysopho-PS; DAG, diacylglycerides; PE, phosphatidylethanolamines; LPE, lyso-PE; PG, phosphatidylglycerols; LPG, lyso-PG, MAG, monoacylglycerides.

**Supplemental Figure 7**

**
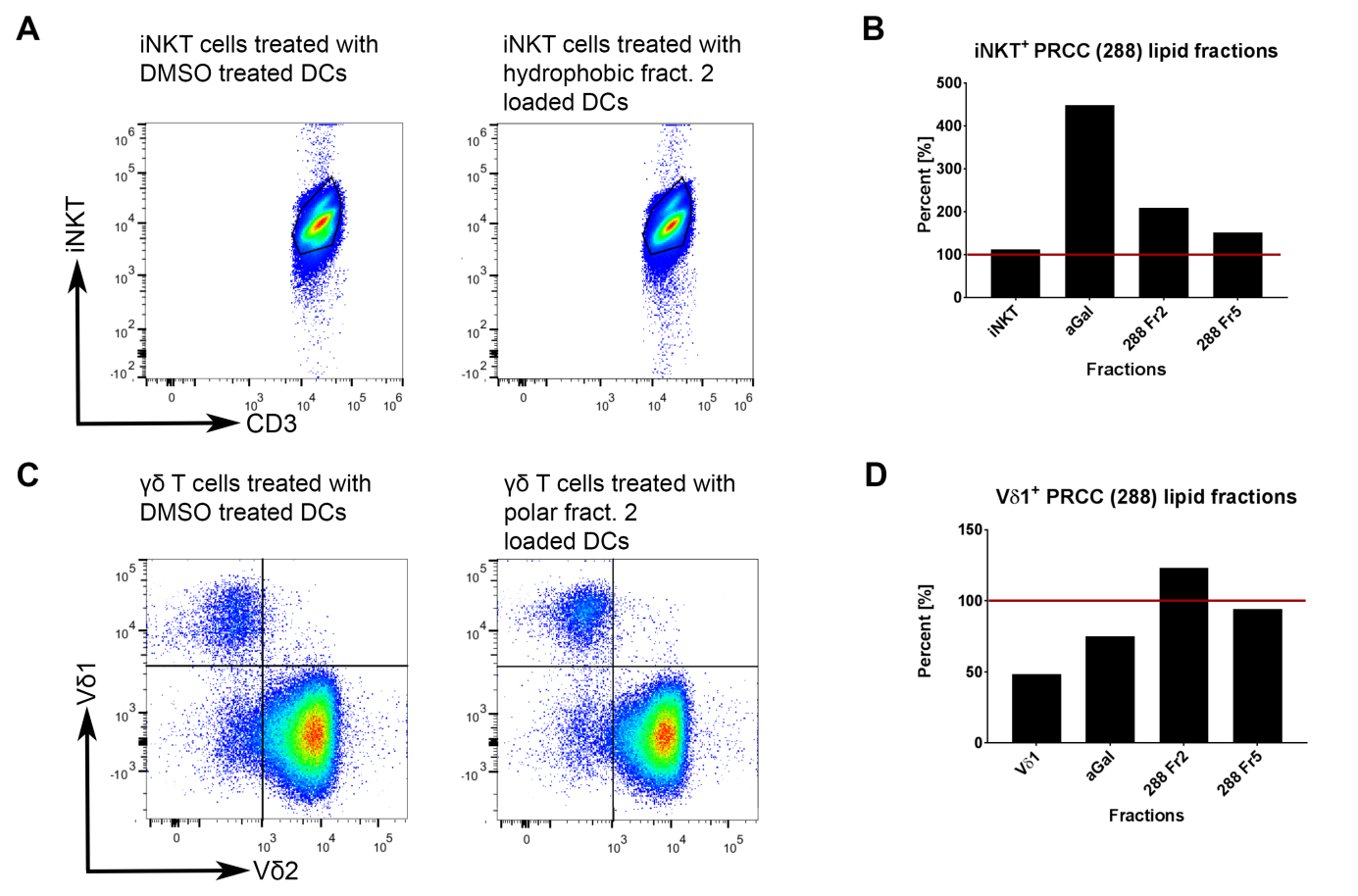
**

**Supplemental figure 7: Tumor own lipids (288) induce proliferation of unconventional T cell populations from healthy donors**

Density plots and quantification of iNKT cells (A, B) and γδ T cells (C, D) co-cultivated with DMSO-treated DCs and DCs loaded with tumor lipid fraction 2. Quantification is shown relative to DMSO control which is set as 100% (red line). DMSO was used as negative control, α-GalCer as positive control. Untreated iNKT and Vδ1 cells are shown to assess whether DMSO treated immature DCs have an effect on the respective T cell population. **(A+B)** Lipid fraction 2 showed the strongest induction of proliferation of healthy donor 3 derived iNKT cells compared to DMSO control (defined as 100%, red line). DMSO has no stimulating effect on iNKT cells. **(C+D)** Lipid fraction 2 is able to induce the proliferation of healthy donor 3 derived Vδ1^+^ T cells compared to DMSO control (defined as 100%, red line). DMSO treated immature DCs has a stimulating effect on Vδ1^+^ T cells.

**Supplemental Figure 8**

**
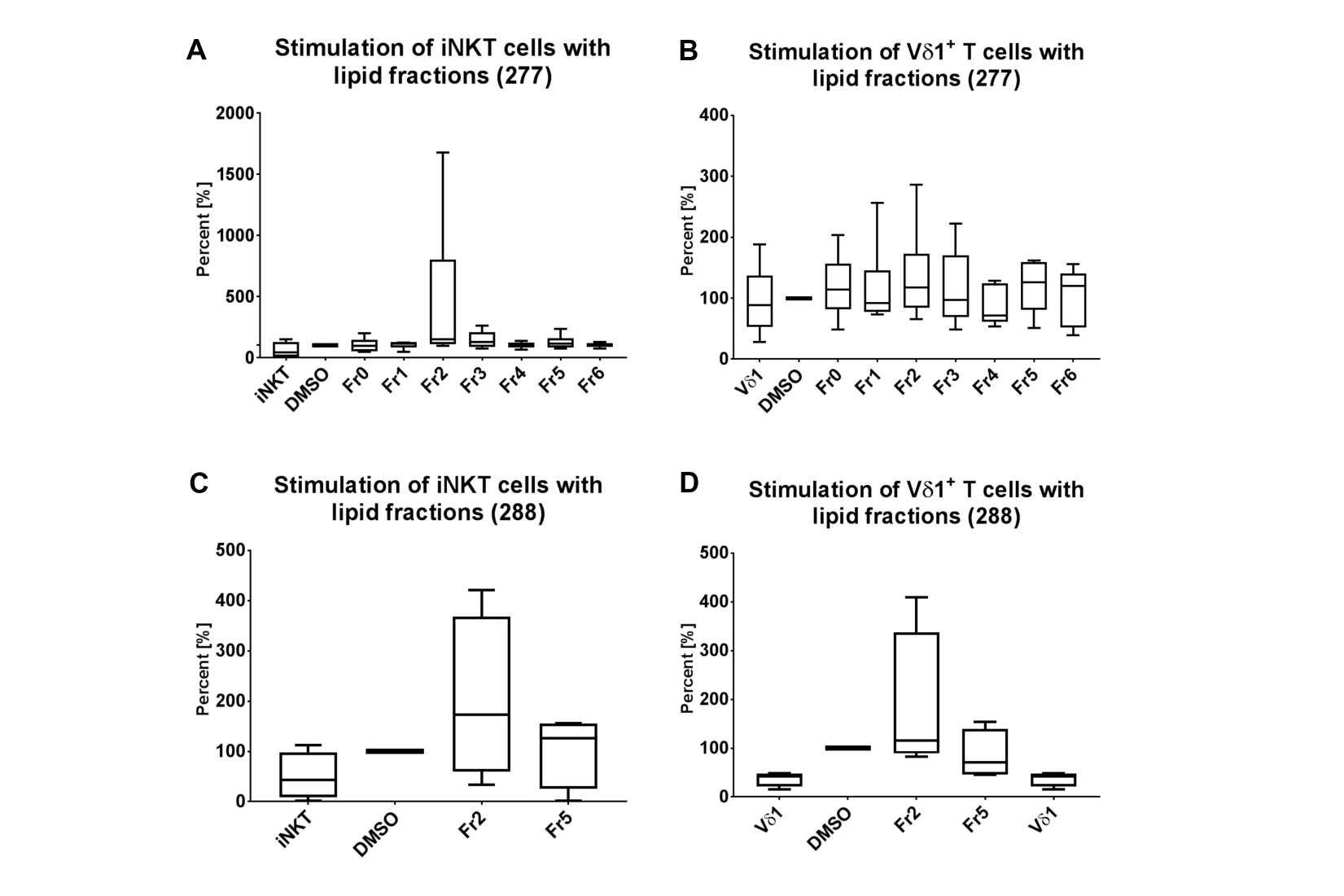
**

**Supplemental figure 8: Proliferation of T cells subsets in response to the stimulation with tumor-derived lipid fractions**

To compare the results of all performed in vitro assays, results of each lipid fraction is shown as boxplot. The % of the proliferation compared to DMSO is shown. **A** and **B** show the results of lipid fractions of patient 277 in 6 healthy donors. **C** and **D** show the results of lipid fractions of patient 277 in 4 healthy donors. Untreated iNKT and Vδ1 cells serve as control to assess whether DMSO-treated immature DCs have an effect on the respective T cell population. Stimulation with tumor own lipid fraction 2 of samples 277 and 288 tends to result in strong proliferation of iNKT cells. Stimulation with tumor own lipid fraction 2 (sample 288) tends to result in strong proliferation of Vδ1 cells. Statistical analyses were performed with the non-parametric Kruskal-Wallis test. There are no significant differences between DMSO and the individual fractions.
